# Coastal ecological impacts from pumice rafts

**DOI:** 10.1038/s41598-022-14614-y

**Published:** 2022-07-19

**Authors:** Yoshikazu Ohno, Akira Iguchi, Mariko Ijima, Ko Yasumoto, Atsushi Suzuki

**Affiliations:** 1 School of Marine Biosciences, Kitasato University, 1-15-1 Kitasato, Minami, Sagamihara, Kanagawa 252-0373 Japan; 2 Geological Survey of Japan, National Institute of Advanced Industrial Science and Technology, 1-1-1 Higashi, Tsukuba, Ibaraki 305-8567 Japan; 3 Research Laboratory on Environmentally Conscious Developments and Technologies [E-Code], National Institute of Advanced Industrial Science and Technology, Tsukuba, Ibaraki 305-8567 Japan

**Keywords:** Environmental impact, Ecosystem ecology

## Abstract

An explosive volcanic eruption occurred in the Ogasawara Islands on 13–15 August 2021, bringing unprecedented amounts of floating pumice to the coast of Okinawa Island in the Ryukyu Archipelago, 1300 km west of the volcano, approximately 2 months later. The coast of Okinawa Island, especially along the northern part, is home to many typical subtropical seascapes, including coral reefs and mangrove forests, so the possible impact of the large amount of pumice is attracting attention. Here, we report early evidence of ecosystem changes as a result of large-scale pumice stranding on coastal beaches, in estuaries and mangrove forests and passage across fringing coral reefs. Massive pumice drifts are major obstacles to fishing activities and ship traffic, but short and long-term changes in coastal ecosystems can also occur. The phenomena observed on Okinawa Island can be a preview of coastal impacts for the Kyushu, Shikoku, Honshu Islands, where pumice has subsequently washed ashore.

## Introduction

**Figure 1:**
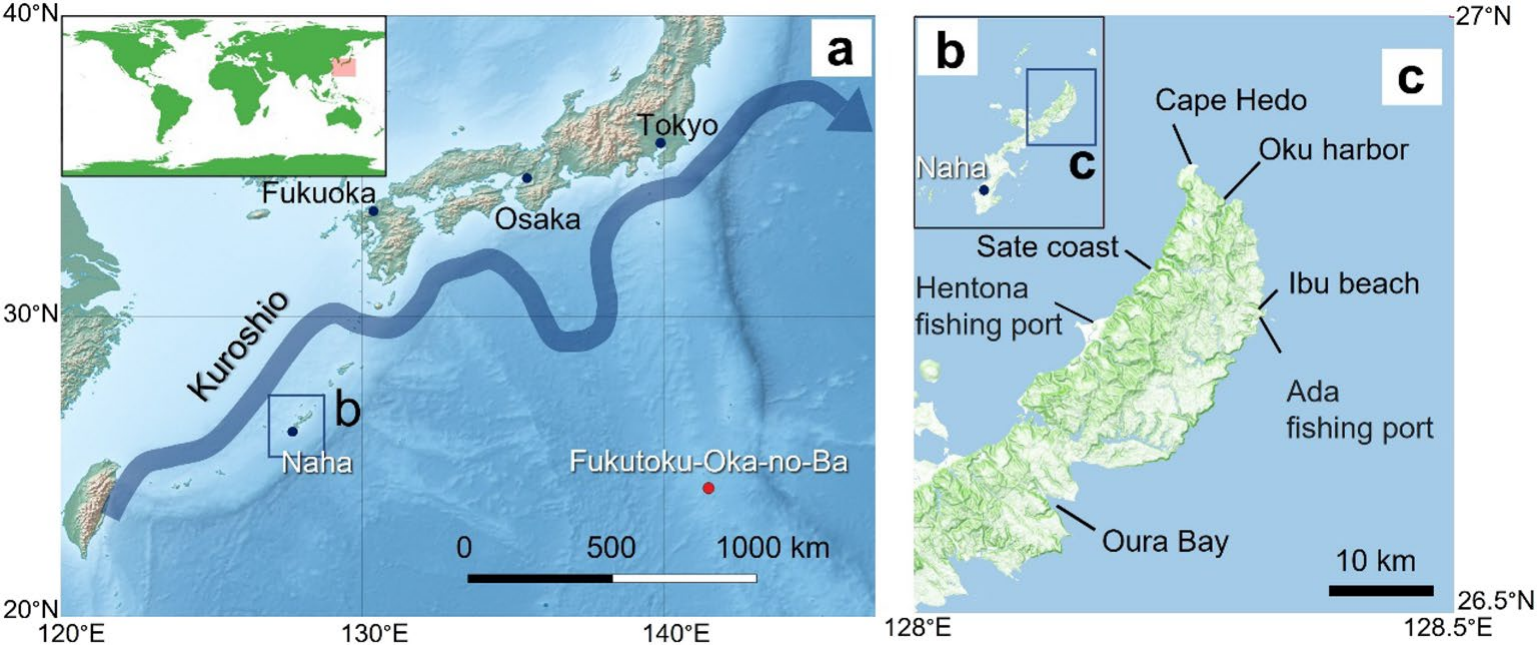
Locations of the eruption site, the island of Okinawa where the pumice drifted, and the observation sites. (**a**) Location of Fukutoku-Oka-no-Ba submarine volcano (red circle) and the Ryukyu Islands, Japan. (**b**) Okinawa Island is ~ 1300 km from the eruption site. (**c**) Sampling sites of drifted pumice along the coast of northern part of Okinawa Island (Kunigami Village) are shown in panel. The flow path of the Kuroshio Current shown in Panel ais based on Quick Bulletin of Ocean Conditions of Japan Coast Guard^12^. Panel is made with Natural Earth (https://www.naturalearthdata.com). Topographic maps of panels b and c are based on GSI Vector Tile Experiment (https://maps.gsi.go.jp/vector/) by the Geospatial Information Authority of Japan.

Pumice rafts can recruit and transport a huge biomass and a wider variety of marine organisms greatly facilitating marine species dispersal^1–6^, but large amounts of floating pumice have the potential to become a natural disaster as a result of human impact^7^. In this paper, we report on the first arrivals of a pumice raft that drifted towards the main island of Okinawa, located in the Southwest Islands of Japan. Okinawa Island is located in the southern region of Japan and has a high level of biodiversity along its coast, including coral reefs, mangroves, and tidal flats^8–10^. Okinawa is influenced by the warm Kuroshio Current (Fig. 1), which flows northward along the west side of the island^11,12^, making the marine environment suitable for tropical and subtropical organisms^8,9,13^. The island therefore has high touristic value, but some areas are threatened by rapidly increasing tourism pressure^14–16^ from ongoing coastal developments^8,13,17^, in parallel with the global changes resulting from multiple human activities^18–20^. In contrast, little coastal development has occurred in the northern part of Okinawa Island, the site of Yambaru National Park, with a high level of biodiversity in the coastal marine environment. In 2021, the region, together with the Yambaru region and Amami Oshima, Tokunoshima, and Iriomote Islands, was named a World Natural Heritage Site^21^.

The Japan Coast Guard reported that a large submarine eruption occurred within the Ogasawara Archipelago (Fukutoku-Oka-no-Ba, Tokyo, Japan: 24.285°N, 141.481°E) on 13 August 2021^22^. Eruption details have been summarized by the Geological Survey of Japan, National Institute of Advanced Industrial Science and Technology^23^, Smithsonian Institution Global Volcanism Network^24^, and Yoshida et al.^25^. Satellite observations were able to capture the formation and then dispersal of the 300-km^2^ pumice raft^26^. Pumice is a highly vesiculated glassy volcanic rock fragment where the vesicularity typically ranges from 50–80% making the pumice have a bulk density less than water. It is commonly light colored, reflecting a high silica content^25^. The large amount of pumice stones produced by this eruption was carried by surface winds and ocean currents for approximately 2 months before reaching the Ryukyu Islands, including the main island of Okinawa, which is located approximately 1300 km away from the volcano (Fig. 1).

In this initial report, we describe the effects of a massive pumice drift on natural systems in the coastal area of northern Okinawa Island (Fig. 1b; Supplementary Table 1) and infer how the ongoing presence of pumice rafts may impact the coastal ecosystem via biological responses to this novel habitat formation. Because of the ever-changing spread of the pumice raft, observations were made at the time and soon after first arrival along the coast of the Yambaru region (Kunigami Village), which is particularly rich ecosystem^21^.

## Results and discussion

### Massive drift of pumice along the northeastern coast of Okinawa Island

**Figure 2:**
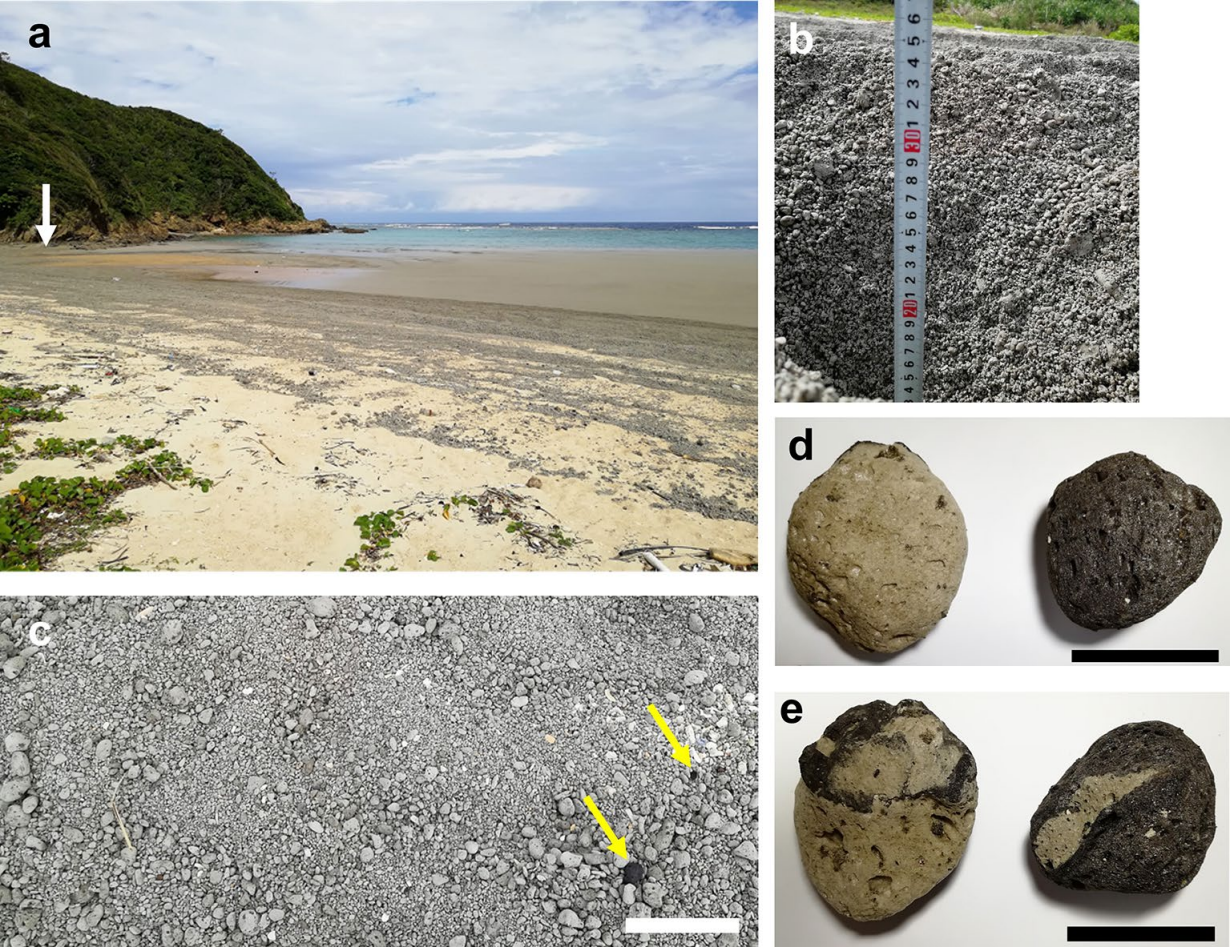
An example of a natural beach on Okinawa Island where pumice has washed ashore. (**a**) Appearance of natural sandy beaches on the northern part of Okinawa Island (Ibu beach, Kunigami Village, 26°75′57.88" N, 128°32′23.32" E). Photo was taken on 24 October 2021. Pumice drifted onto the sandy beach and formed a striped pattern. The white-capped waves indicate on the place where the reef edge exist. The white arrow points to the mangrove river estuary corresponding to Fig. 9. (**b**) Estimation of the pumice sedimentation depth on the original sand beach surface. (**c**) The high tide zone of the natural sandy beach is covered with pumice pebbles and stones. Yellow arrows indicate black pumice stones. Scale bar: 10 cm. (**d**, **e**) Front and back of examples of relatively large pumice stones from the same beach. The left image is mostly light brown, whereas the right image is almost black. Scale bars: 5 cm.

**Figure 3:**
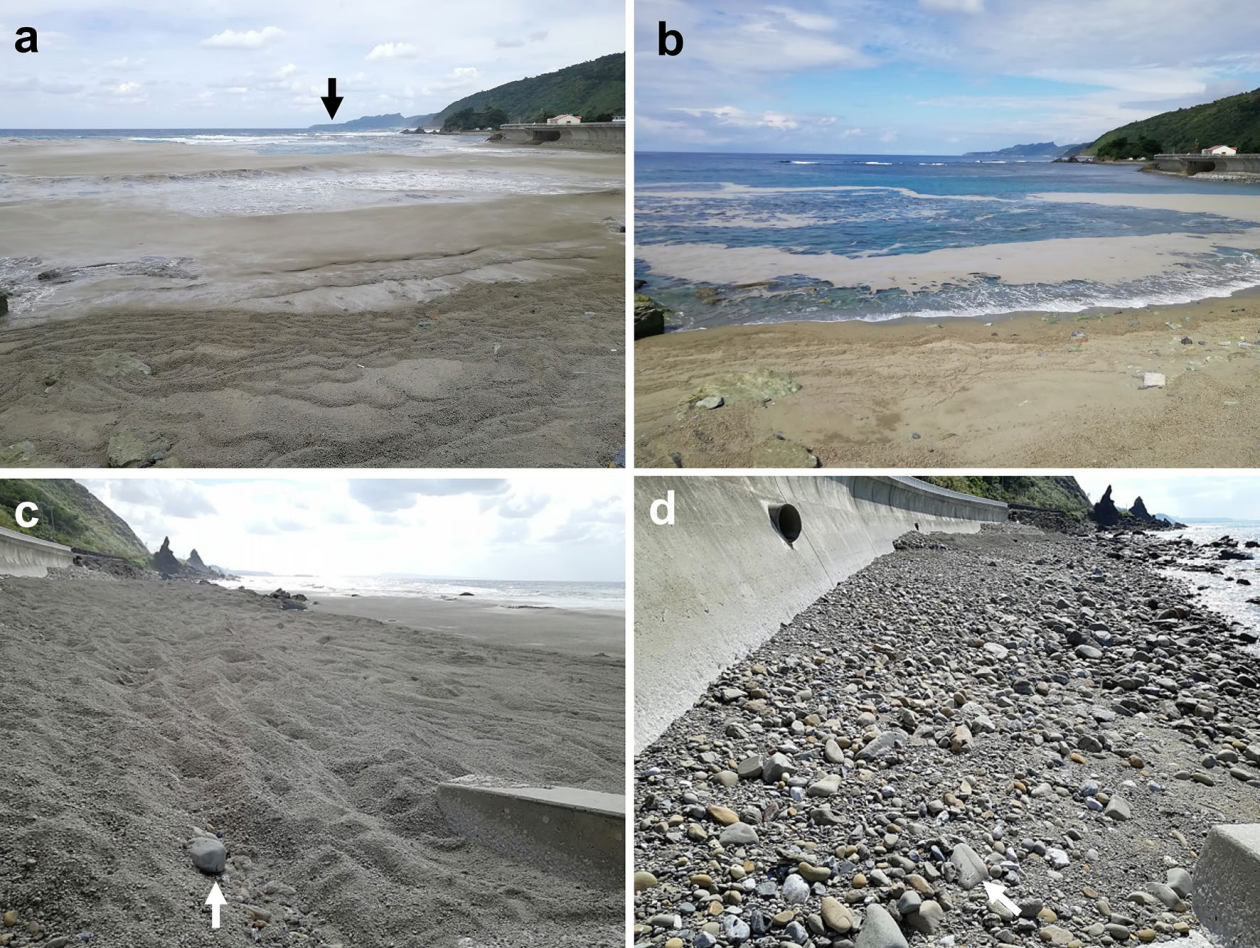
Short-term migration of pumice from beaches as revealed by stationary observations. These four photos were taken at two sites on northern Okinawa Island on two consecutive days, 23 and 24 October 2021. (**a**, **b**) A sandy beach along the Sate Coast (26°78′84.56″ N, 128°22′30.57″ E). It was windy on the first day, and pumice stones were washed up with the waves. Almost all the pumice stones were removed from the beach and transported offshore on the following day. The black arrow in photo (a) indicates Cape Hedo, the northernmost tip of Okinawa Island. (**c**, **d**) At this gravelly beach (26°80′83.25″ N, 128°23′38.56″ E), pumice fully covers the seawall on the first day, but all of the pumice stones washed away, leaving the original gravels, on the following day. The white arrow in each photo indicates an identical marker stone placed on the beach. Weather data of northern Okinawa (https://www.data.jma.go.jp/obd/stats/etrn/view/daily_a1.php?prec_no=91&block_no=0901&year=2021&month=10&day=23&view=g_wsp) and tidal data (Naha: 26°13′ N, 127°40′ E) (https://www.data.jma.go.jp/gmd/kaiyou/db/tide/genbo/genbo.php) are provided by Japan Meteorological Agency.

**Figure 4:**
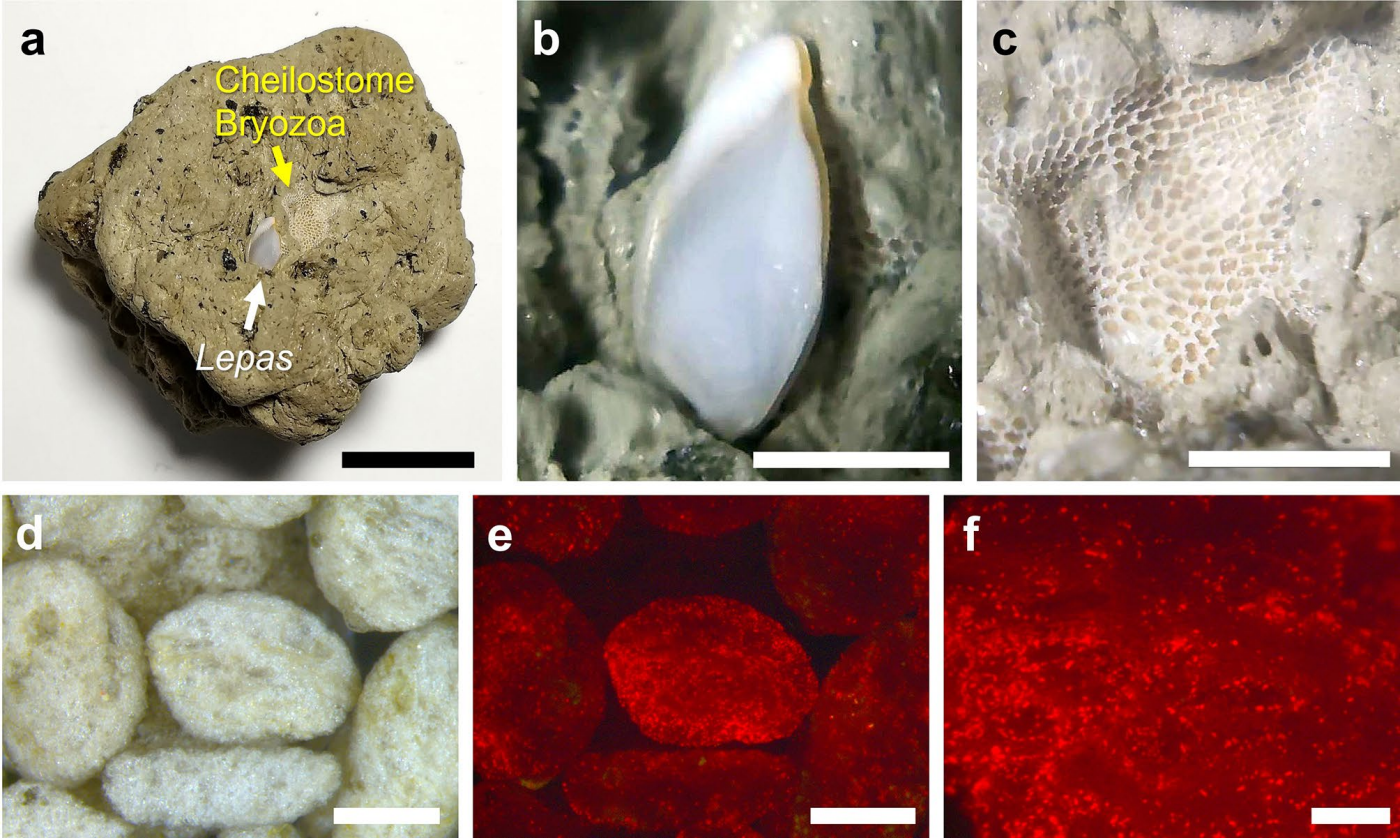
Pumice stones settled by marine organisms. (**a**) Pumice collected from Ibu beach on 31 October 2021. Two marine benthos coexist close together on a pumice stone. Scale bar: 1 cm. (**b**) Enlarged image of the *Lepas* barnacle. Scale bar: 3 mm. (**c**) Enlarged image of the bryozoan. Scale bar: 3 mm. (**d**) Stereo microscopic image of pumice pebbles of a few millimeters in diameter collected from Ibu beach on 15 January 2022. The light brown coloration indicates some algal/cyanobacterial growth on the pumice. Scale bar 1 mm. (**e**) Red autofluorescence was detected from pumice pebbles. Image corresponds to (d). Autofluorescence from microalgae was confirmed by Supplementary Fig. 2. Scale bar 1 mm. (**f**) Enlarged image of the center of the figure of (e) shows red autofluorescent signals with a diameter of 10–30 µm. Scale bar: 200 µm.

A large amount of pumice stones reached and was deposited along the northeastern coast of Okinawa Island, that were brought by strong seasonal northeasterly winds (Supplementary Video 1). The pumice was thought to be brought by the Kuroshio countercurrent from sites near the Ogasawara Archipelago 1300 km away. Because the Kuroshio countercurrent is composed of various medium-sized eddies in the ocean, the current does not always flow in one direction and as a continuous flow^27,28^. The pumice drift was more strongly controlled by the seasonal northwesterly winds to be transported to Okinawa across the Philippine Sea (Fig. 1a). The pumice raft reached the northern part of Okinawa approximately 2 months after the eruption (Figs. 2, 3 and 4). According to a very recent report, the pumice clasts were drifting ashore in Thailand (traveling 4000 km-long distance) across the South China Sea within half a year of this eruption^29^. Most pumice stones were gray, but some pumice was banded, and others were black reflecting some compositional variation^25,29^ (Fig. 2d,e). The Kuroshio Current is faster than the Kuroshio countercurrent^27^, so some pumice clasts have already reached the main island of Japan^25^. Tracking the dispersal of the pumice will allow a better forecasting model based on observed raft trajectories by considering exact wind effects in the Philippine Sea^30^.

### Changes in the coastal landscape: natural beaches and estuaries

Marine calcifiers, including corals, calcareous algae, and foraminifers, produce white sandy beaches on Okinawa Island. However, the gray pumice drifting ashore changed the white sand beach, especially along the northeastern coastline. We observed several lines of pumice aggregations, suggesting that pumice was brought ashore by wavefronts several times produced by a strong north wind at the tide lines (Supplementary Video 1; Fig. 2a). At the same sampling site, the thickest depth of beached pumice was more than 30 cm (Fig. 2b; Supplementary Video 2). Most of the pumice stones were from 0.5 cm to 3 cm in diameter, with a few black pumice stones included (Fig. 2c: yellow arrow). Pumice stones arrived at the estuaries of some brackish rivers (Fig. 8, Supplementary Fig. 1a) and mangrove forests in northwest Okinawa (Fig. 9).

Pumice stones and pumice rafts show dynamic behavior in a short period. We captured photographs 24 h apart at two positions on the shore of Okinawa, which allowed us to compare the pumice dynamics during this period (Fig. 3). Within that time frame, there were two high tides, and the tide level changed by up to 170 cm. As seen in Fig. 3a, on the first day, the coast was covered with pumice, and floating pumice could be seen on the seafront. The north wind was strong that day, as shown by the relatively high waves near the shore as well as white‐crested waves near the reef edge. By the following day, most of the pumice had been moved offshore by tides and winds (Fig. 3b), indicating that newly beached pumice raft deposits were removed quickly from open beach areas. At another site on a gravelly beach, pumice fully covered the seawall on the first day, but almost all of the pumice stones were washed away, leaving the original gravels, on the following day (Fig. 3c,d). Japan Meteorological Agency (Oku station: 232 m above sea level, latitude 26°50.1, longitude 128°16.3') reported that northerly winds were blowing (mean wind speed: 3.4 m/s) on 23rd October in northern Okinawa. The following day, the wind direction changed to the east-southeast; blowing offshore (mean wind speed: 2.9 m/s), resulting in the dramatic removal of pumice form the coast (Fig. 3). These observations indicate that surface winds rather than ocean currents had a strong influence on the raft trajectory and residence time on beaches, and are consistent with past research^5^. These observations lead us to expect that the pumice rafts will disappear from the coast of Okinawa fairly quickly, but in fact, there have been many cases where they have come back again in a few days. Although the overall amount of pumice drifting has been decreasing, a small amount of pumice has been drifting in coastal area of Okinawa in May, 2022^31^. It is unlikely that large amounts of pumice will drift repeatedly throughout Okinawa Prefecture as reported in this report, but it should be noted that detached pumice material remains in beach and river runoff.

### Biofouling of sessile organisms on pumice arriving to Okinawa

It is noteworthy that the pumice rafts traveled over the deep Philippine Sea for over 2 months, and on arrival in Okinawa there was little to no biofouling of the pumice (Fig. 2). Some stranded pumices observed on Okinawa beaches had become habitats for sessile organisms (Fig. 4), as reported in previous studies^1–6,29^. Goose barnacles (*Lepas* sp.) without external damage to the shell were the most abundant species observed on the pumice (Fig. 4b). *Lepas* is a common biofouling taxon distributed globally and plays a role in biofouling as a foundation organism. The shell growth rate is more than 1 mm/day in some *Lepas* species^32^ suggesting that the *Lepas* had been growing on the pumice for about two weeks. Measurements of the shell size of *Lepas* attached to the pumice collections conducted in the same area (Supplementary Video 2) showed a bias toward larger sizes in the second collection (5.92 ± 3.86 mm (average ± S.D.), n = 75, 13 November 2021) than in the first one (3.43 ± 1.08 mm, n = 21, 31 October 2021), and significant differences were detected between the measurement periods (Mann–Whitney U test, *p* < 0.05). These data imply that the barnacles settled on the pumice stones and started to grow near Okinawa. The shell size would be larger if the barnacles had settled and grown on the surface of the pumice near the Ogasawara Islands as because the travel time was at least 50 days to Okinawa. The pumice raft crossed deep ocean water of the Philippine Sea from the source volcano and had no island or reef encounters on the way; it therefore could not recruit shallow marine organisms like *Lepas* early. This is a big difference to the pumice rafts in the Southwest Pacific^1,5,6^. A cheilostome bryozoan was also found on the same pumice sample (Fig. 4c). Bryozoans are colonial marine invertebrates that construct an exoskeleton composed of aragonite and calcite^33,34^.

Red autofluorescence was detected from the pumice surface (Fig. 4d), thought to be derived from the chlorophyll of microalgae. After treating with acetone and methanol, autofluorescence from pumice dramatically disappeared (see Supplementary Fig. 2) with a diameter of several tens of micrometers (Fig. 4e, f). The texture of the pumice clasts (Fig. 4d) and the bright spots of the red fluorescent signal (Fig. 4e) were not completely coincidental. Red signals are more prominent in the vesicle depressions rather than on the outer surfaces where abrasion would occur. As the pumice pebbles in the rafts are constantly rubbed and worn, only microorganisms are likely to survive on the surface on small pumice pebbles. Once deposited in estuaries and in brackish water, filamentous algae quickly grew on the pumice (Supplementary Fig. 3). Our study did not carry out species identification of microorganisms on pumice stones. However, Naya & Hatanaka^35^ already discussed a possibility that pumice rafts may contribute to the dispersal of some attached marine diatoms.

Genetic studies have revealed the transport of larval corals^36^ and crown-of-thorn starfish^37^ between the Okinawa and Ogasawara Archipelagos, which are more than 1000 km apart, with no large islands in between. Given that corals can be transported long-distances on pumice^1–3,5,6^, integrating spatiotemporal information on the exact pumice movement on the sea surface with such genetic analyses may help to clarify the dispersal processes of marine organisms in greater detail.

Considering that small sessile organisms were often found on pumice rafts (Fig. 4), it is easy to imagine that pumice rafts transport not only multicellular organisms but also microorganisms such as bacteria. Some astrobiology studies have proposed that pumice might have functioned as a habitat for the earliest settlements of microorganisms^38,39^. Considering this study, pumice rafts may serve a variety of functions in bacterial ecosystems, such as connecting populations over long distances^1–6^ and serving as a direct link between sandy beaches and the ocean (Fig. 3). Pumice has a large surface area with many vesicles^40–42^. When pumice stones are impacted onto solid surfaces such as by wave action, they may break apart, thus enlarging the surface area and leading to a dramatic increase in available bacterial habitat; pumice may continue to serve as an ecosystem mediator for a long period of time.

### Impacts on fishes and other organisms in coastal waters

**Figure 5:**
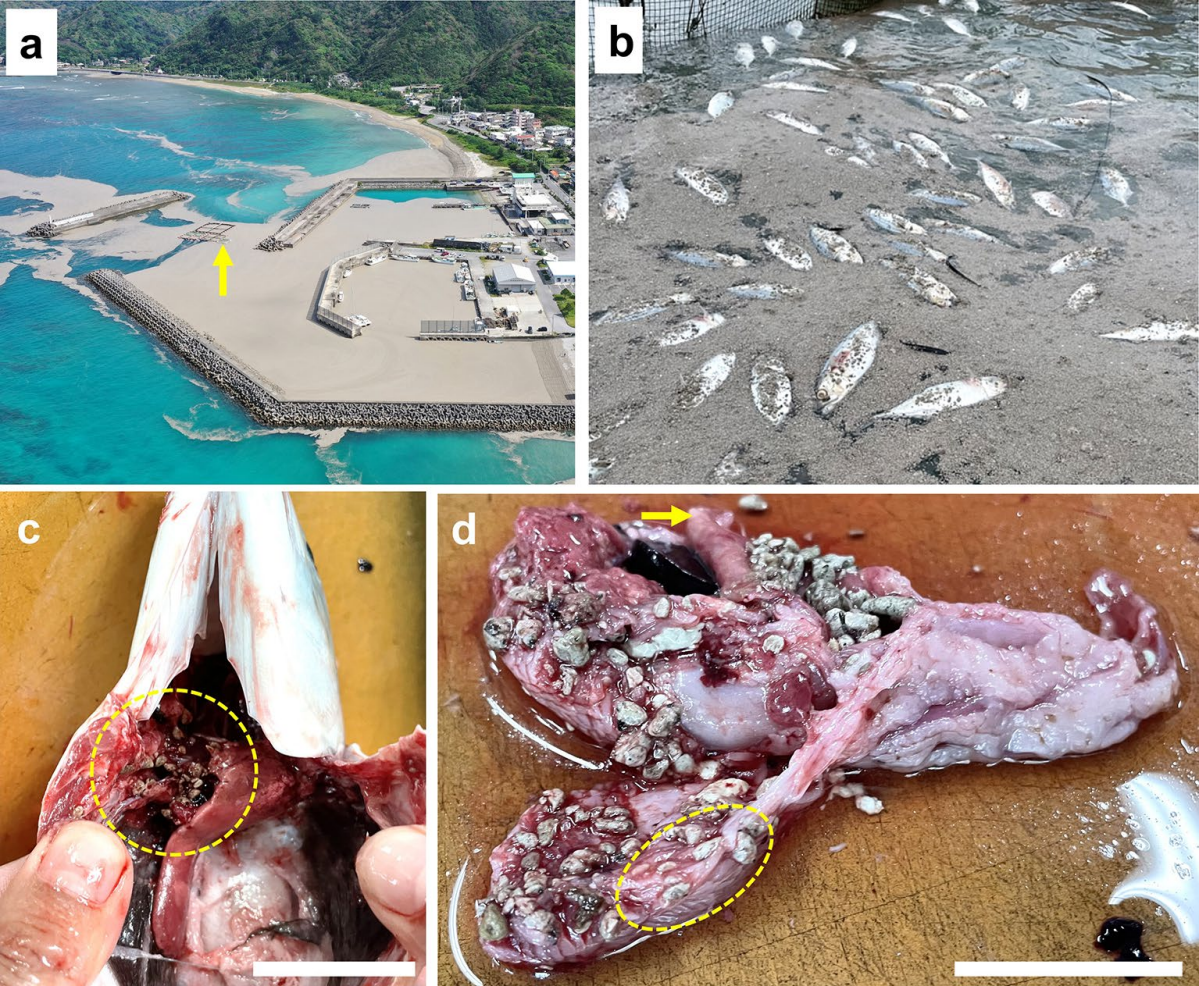
Large quantities of pumice drifted ashore, killing farmed Indian mackerel (*Rastrelliger kanagurta*). (**a**) Aerial image taken by a drone at the Hentona fishing harbor on 24 October 2021; yellow arrow points to the fish cage adjacent to the bay covered with pumice rafts (Hentona fishing harbor: 26°74′83.84″ N, 128°17′76.33″ E). (**b**) A large amount of pumice washed ashore and cultured fish died in the fish cages. The local fishermen association estimated that more than 200 individuals had died by early November. (**c**) When fish were opened, they had swallowed many pumice pebbles (yellow enclosed area: esophagus). (**d**) The appearance of the stomach to the intestines is shown. The yellow arrow indicates the part of the fish from the esophagus to the stomach. The appearance of the intestine is craggy due to the pumice pebbles stuck in the intestine. In the yellow circle, the pebbles in the intestine were transparent and partially came out from it.

One portion of the pumice raft reached the Hentona fishing port (Fig. 5a), where more than 200 farmed Indian mackerel (*Rastrelliger kanagurta*) had died in the fishery cages in the bay by early November (Fig. 5b). Fish stomachs were filled with pumice stones (Fig. 5c), suggesting that they had confused pumice for food. The digestive system in the fish is filled with numerous pumice stones, and in places, the pumice stones are visible through the intestinal wall or are damaged, suggesting that the direct cause of death of the farmed fishes was not starvation but damage to the fish's digestive tissues. This species of fish is a filter feeder swimming with its mouth open while feeding. The same feeding behaviour is also seen in the fin whale (*Balaenoptera physalus*) and basking shark (*Cetorhinus maximus*), two marine species studied with regard to environmental pollution by microplastic debris^43^. When marine wildlife such as turtles, seabirds, and whales mistake floating plastic waste for prey, most die of starvation, as their stomachs become filled with plastic debris^44,45^. We are concerned that a similar situation may occur with filter-feeding fishes mistakenly consuming pumice stones.

Some migratory fishes must swim with their mouths open and breathe by constantly taking oxygen dissolved in the seawater passing through their gills (e.g., tuna, bonito, yellowtail, sardine, mackerel, swordfish and basking shark). If pumice pebbles or particles were to pass through the gills, depending on clast size, they may physically damage the tissue. This damage may affect the survival of these economically important fishes and perhaps alter their population dynamics. Thus far, it is unclear how the catches in the seas around Okinawa have changed since the arrival of the drifting pumice. If this hypothesis is correct, then pumice traveling on the Kuroshio Current could also affect fisheries in the seas around Japan. Through more research, the impact of large quantities of pumice on marine life will become clearer in the future.

Pumice rafts may also affect the upriver migration of fishes^46,47^, which move between the sea and rivers according to their life histories. Pumice covered the brackish estuary of the Oku River in northwest Okinawa (Oku River, Supplementary Fig. 1a), adjacent to the Oku harbor (Supplementary Fig. 1b). We observed that pumice stones covered the river bottom as well as mangrove mudflats. Further studies are needed to assess the impacts of pumice rafts on anadromous and amphidromous fish populations.

### Coral reef interactions

**Figure 6:**
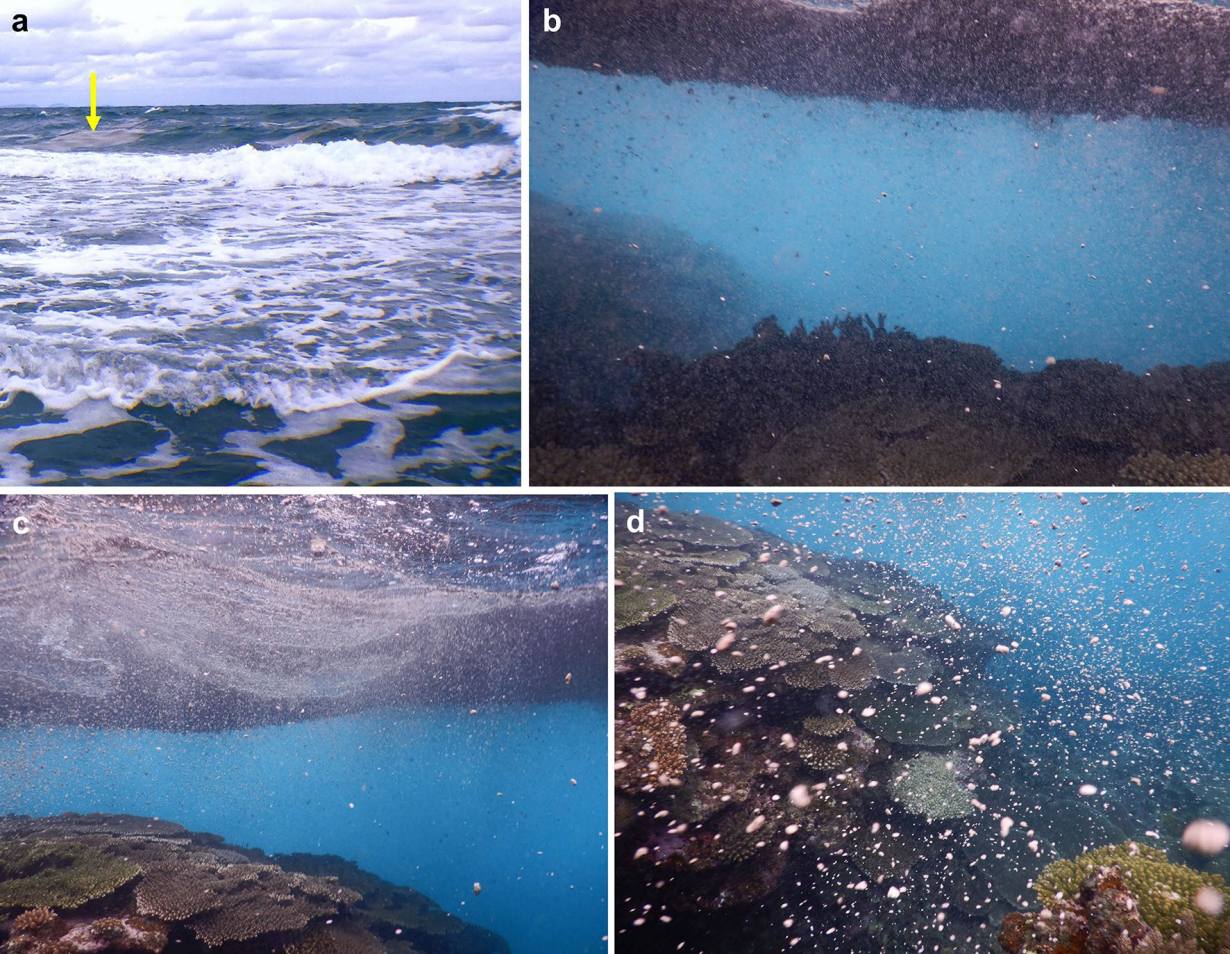
Pumice raft shading corals at the reef edge. Pumice rafts shading corals at the reef-edge (Iji coastal area: 26°46′00.2″ N, 128°11′35.3"). Photos were taken at low tide during the day on 14 November 2021, and the pumice raft floated approximately 50 cm from the top of the reef (see Supplementary Video 3). (**a**) View of the reef edge from the intertidal zone at low tide. The yellow arrow indicates that the pumice raft was drifting across the reef edge. (**b**) It was dark under the pumice rafts. (**c**) The pumice rafts were shown to disperse as the waves came in, allowing sunlight to reach the coral through their gaps. (**d**) The waves crashed the pumice rafts, causing countless pumice stones to drift fast through the water.

Coral reefs are diverse ecosystems and provide coastal protection from waves^48^. Reef-building corals maintain photosynthetic algae (zooxanthellae) that live in their tissues and play a critical role in supplying the coral with glucose, glycerol, and amino acids, which are the products of photosynthesis under energetic consequences of flexible symbiont associations (i.e., mutualistic relationships)^49^. If the symbiotic relationship breaks down, the coral tissue will turn white (bleaching), and in the worst case, the coral will die^50,51^. Besides, pumice rafts may provide the opportunity for any attached species to escape from local unfavorable conditions. Previous studies have shown that pumice rafts contribute to coral dispersal^1–3,5,6^. But potential long-term impacts of pumice rafts on coral reefs remain unknown. At Okinawa, pumice rafts washed ashore in inland bays such as beaches (Figs. 2, 3) and concentrated in harbors (Fig. 5, Supplementary Fig. 1b,c), but such areas are not often inhabited by corals. Where pumice rafts were observed to pass over fringing coral reefs, sunlight was temporarily reduced (Fig. 6b). No sustained coverage of the fringing reefs was observed to indicate any photosynthesis was inhibited, causing negative effects for coral physiology.

**Figure 7:**
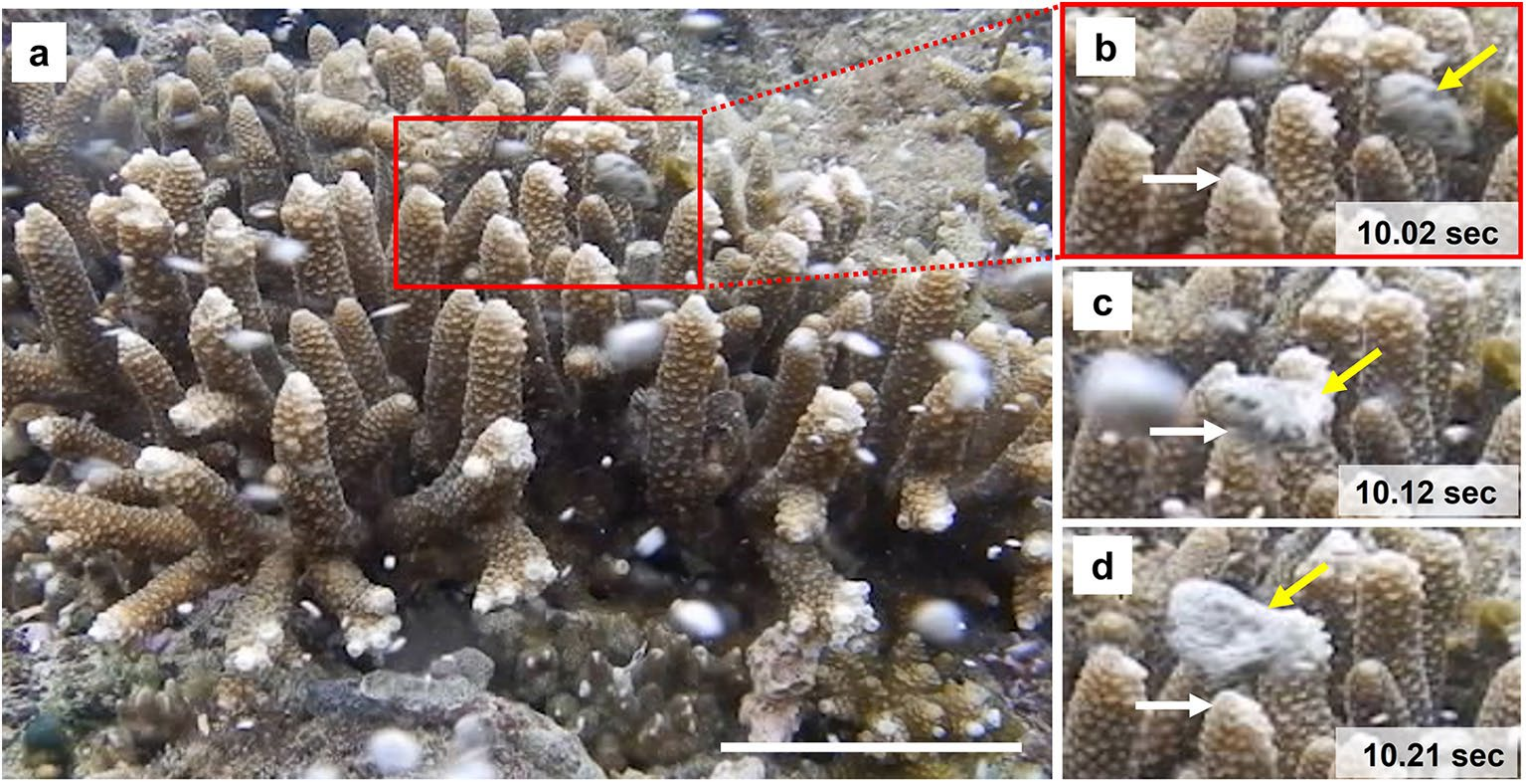
Underwater photography showing that pumice stones hit coral branches. Underwater photographs showing the possible damage of corals by the ablation of pumice stones in a shallow coral reef environment. (**a**) Pumice pebbles are shown drifting around the coral and driven fast by wave action. This figure corresponds to Supplementary Video 4. Scale bar: 5 cm. (**b**–**d**) A pumice stone with a long axis of approximately 2 cm (yellow arrow) contacted the tip of a coral branch (white arrow) and changed its direction within a short period. The images were taken less than 50 cm below the sea surface. These photos were taken on 14 November 2021.

Locally, small pumice pieces occasionally hit shallow corals in response to breaking waves across the fringing reef (Fig. 7, Supplementary Video 4). In larger pumice rafts and containing larger pieces of pumice, a potential exists for surface damage and stress to corals by pumice impacts. Reef-building corals have a fragile layer of soft tissue on a calcium carbonate skeleton. To protect and defend themselves from various kinds of foreign matter in the environment (e.g., sand and bacteria), coral tissues continuously produce mucus, which is thought to play a role in coral halobiont defense, possibly through the production of antimicrobial substances^52^. Microscopic scratches may be caused by drifting pumice stones, which could induce inner tissue exposure and the loss of mucus function. This situation may lead to infection of the coral surface by bacteria and other pathogens if adhered to the pumice stones. Previous studies have reported that coral tissue can be lost in response to mechanical stress leading to the induction of coral diseases^16^, and multiple environmental stressors could result in the expansion of harmful bacteria in the reef environment^53,54^. Currently, the exact impact of the pumice on the coral reef ecosystems around Okinawa is unclear, but the systems should be closely monitored.

### Impact on mangrove ecosystems caused by sudden changes in sediment properties

In the tropics and subtropics, mangrove ecosystems play critical roles in interactions between land and sea. In fact, mangrove ecosystems are linked to neighboring environments such as coral reefs and seagrass beds through the movement of organisms and the circulation of materials^55,56^. Recent scientific advances have clarified the microbial environment of mangroves by using microarray-based genomic technology for detecting functional genes. Their unique microbiota suggests that they may perform several important functions, such as recycling nutrients, destroying pollutants, and treating anthropogenic wastes. Many antibiotic and metal resistance functional genes are detected^57^.

**Figure 8:**
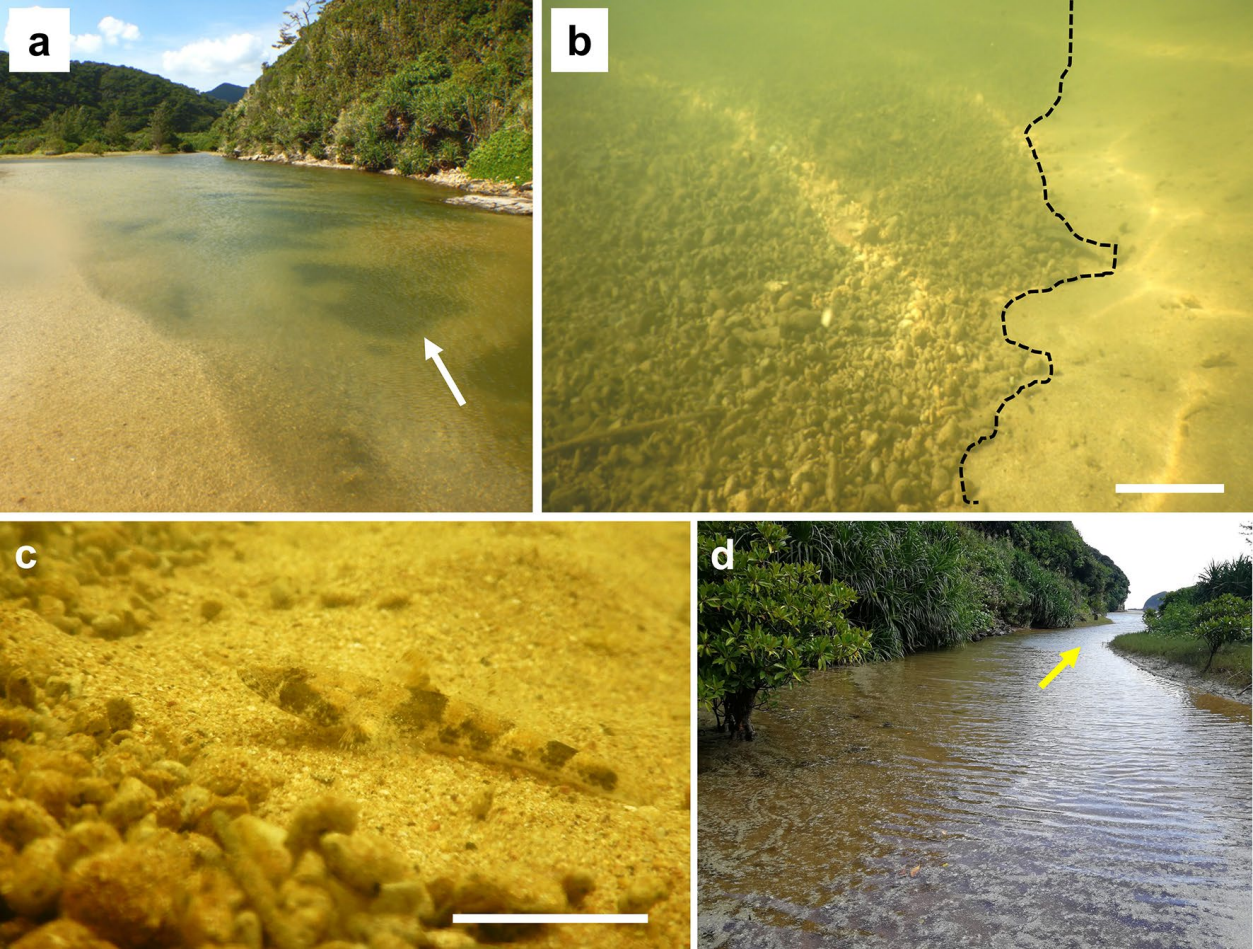
Pumice has begun to settle onto the bed of the brackish river. (**a**) In the lower reaches of the Ibu River (26°75′71.47″ N, 128°31′97.47" E), connecting to the coast north of the Ibu beach. Photo taken from the estuary side at low tide. The white arrow points to a dark gray aggregation of pumice on the bottom of the river. (**b**) Enlarged image of (a). The black dotted line marks the boundary between the pumice stones (left) and the original sandy river bottom (right). Supplementary Video 5, showing pumice stones swaying in the water, was recorded in this area. Scale bar: 10 cm. (**c**) Psammogobius biocellatus can be seen at the river bottom. Pumice stones were located left on this image. (**d**) A little further up the river, where mangroves can be seen to the left. Yellow arrow points toward the estuary. These photos were taken on 13 November 2021. At the same location, filamentous algae were growing rapidly on the pumice surface in mid-January (see Supplementary Fig. 3).

More protracted deposition of pumice stones occurred in estuaries around Okinawa characterized by mangroves (Fig. 8). A river flowing through a mangrove forest connects to Ibu Beach (Fig. 2a: white arrow points to the river mouth). In the brackish water of the river, approximately 100 m from the mouth, many pumice stones were found to have sunk (Fig. 8, 9a). The pumice stones were easily moved by hand, suggesting that the pumice had not completely lost its buoyancy, and some of the pumice stones at the bottom of the river swayed slowly in the water flow (Supplementary Video 5). For a goby (*Psammogobius biocellatus*) on the river bottom seemed to have already acclimatized to the environment, at the boundary between the pumice stones and conventional sediment (Fig. 8c). Further up the river (approximately 200 m from the river mouth), floating pumice stones reached the point where orange mangrove (*Bruguiera gymnorhiza*) trees were growing (Fig. 8d). There was no heavy rainfall during the field observation period. If heavy rains occur and the flow rate of the river increases, however, pumice stones could be cleaned out of the estuaries and be transported back to the ocean. It will be necessary to monitor river substrate changes and to assess how pumice might affect river and estuary ecosystems (e.g., fish migration) in the future^46,47^. Within four months of the massive pumice adrift to Okinawa Island, we could easily observe green filamentous algae sprout from pumice pebbles (Supplementary Fig. 3). Our observations are consistent with those already reported in previous studies^2,4–6^.

**Figure 9:**
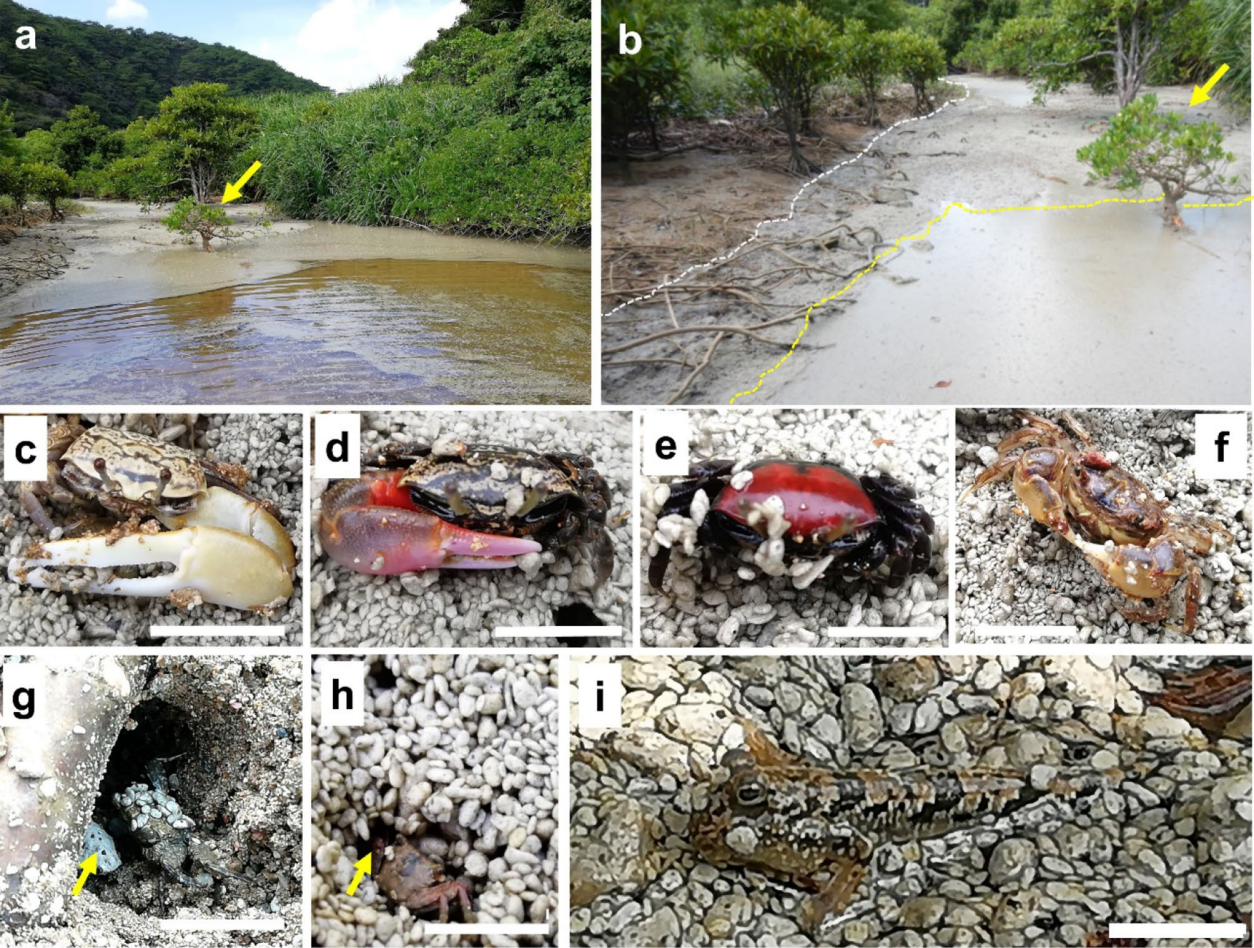
Behavior of mangrove organisms affected by drifting pumice. (**a**) Pumice raft adrift in a mangrove river (26°75′72.51″ N, 128°31′89.44" E), where a colony of *Kandelia obovata* (yellow arrow) and *Bruguiera gymnorhiza* trees coexist. (**b**) Enlarged image from (a), with the yellow arrow pointing to the same tree. The white dotted line indicates the boundary between the original mudflats and pumice-covered substrates, and the yellow dotted line marks the border of the water. (**c**) A fiddler crab (*Uca lactea lactea*) on the pumice pebbles. Video recording of the *U. lactea.* Behavior on the pumice-covered sediment shows the crab's inability to return to its burrow, which had collapsed due to the pumice stones (Supplementary Video 6), and the competition between the species to acquire the burrow (Supplementary Video 7). Scale bar: 1 cm. (**d**) A fiddler crab (*Uca coaractata*) on the pumice pebbles. Scale bar: 1 cm. (**e**) A fiddler crab (*Uca chlorophthalma crassipes*) on the pumice covering mudflat. Scale bar: 1 cm. (**f**) A mitten crab (*helical epicure*) on the pumice substrate. Scale bar: 1 cm. (**g**) A sesarmid crab that cannot go back into its burrow because a pumice stone has blocked the entrance (yellow arrow); its body surface is covered with pumice grains. Scale bar: 1 cm. (**h**) A sesarmid crab tries to hide in a depression near the burrow; it is unable to hide in its burrow, which is blocked by pumice (red arrow). (**i**) A mudskipper (*Periophthalmus argentilineatus*) was placed on the pumice-covered water surface. It seemed difficult for fish to jump across the pumice-covered surface (Supplementary Video 8). Scale bar: 1 cm.

Pumice rafts also drifted onto the mangrove mudflats (Fig. 9a), which were almost completely covered with pumice pebbles (Fig. 9b). Although this area is covered with a massive amount of pumice, many of the crabs and gobies survived under these circumstances (Fig. 9c–i), and we did not find any evidence of mass die-offs in the pumice-covered mangrove at the beginning of this study period. However, the surface layer of the crab burrow was covered with pumice and was prone to collapse; in some cases, the crab burrows were blocked (Fig. 9g). Observations of behavior, with particular focus on a fiddler crab (*Uca lactea lacteal*) in Fig. 9c, showed that in some cases, it had given up trying to enter the burrow, which easily collapsed under the pumice stones (Supplementary Video 6). In another case, competition to acquire burrows emerged, making it difficult for smaller individuals to obtain a burrow even within the same species (Supplementary Video 7). The pumice-covered substratum is different from the original mud substratum, making it a particularly difficult environment for small crabs. To adapt to this environment, small crabs have already shown alternative behaviors, such as hiding in the spaces among pumice pebbles (Fig. 9h). Fiddler crabs use their claws to put substrates in their mouth and then sift through the substrate and eat the organic matter (e.g., algae, fungi, and tiny insects)^58^. As the substrate has been replaced by pumice instead of sand, feeding behavior may be inhibited, especially for smaller individuals. If pumice stones occupy a large surface area during the breeding season, however, they may interfere with the spawning and larval dispersal of these crabs. Three months after we started the survey, we could hardly observe the burrows of the fiddler crab or their population on the pumice cover mudflat. It is speculated that the environment where the fiddler crabs live was covered with pumice pebbles, hindering eating behavior, with the younger individuals being more affected.

Another observed mangrove inhabitant is the mudskipper (*Periophthalmus argentilineatus*), a fish that jumps on the surface of the water around a creek near the riverbank. However, when the pumice grains stuck to the fish’s body, they did not move well and appeared to sink (Supplementary Video 8). The mudskipper has relatively thin skin that is suitable for life on land and breathing oxygen^59^, such that fish movement may result in the pumice on its skin surface causing microinjuries. A recent molecular study suggested that the expansion of innate immune system genes in mudskippers may provide a defense against terrestrial pathogens^60^. Because the bacterial composition of the sediment may also be changed by drifting pumice stones, the immune response of this fish may be altered if the pumice pebbles remain on the mudflats for a long time. We are also concerned about mudskippers’ feeding activities as well as territorial and courtship behavior on pumice-covered mangroves^61^. In addition, the mudskipper has a special egg-laying behavior: the fish deposits eggs on the walls of an air-filled chamber within its burrow to provide air to the eggs in the lower oxygen conditions in the mud^62^. Likewise, the various behavioral patterns of gobies could also be affected by the drift of pumice in the mangrove. In addition to mangrove fishes, we assume that drifting pumice stones may especially affect fish communities inhabiting soft-sediment coastal areas where pumice pebbles easily sink or bury the soft sediment^10^.

As described above, the organisms living in the mangrove tidal flat have difficulty finding shelter due to the change in the substrate (Fig. 9c–i, Supplementary Videos 6–8). Thus, it is likely to be preyed upon by other wild animals, such as the Okinawa rail (*Hypotaenidia okinawae*). *H. okinawae* is a flightless rail that is declared the National Natural Treasure (Agency for Cultural Affairs) and endemic to Yambaru region (Supplementary Videos 9). The pumice-covered mangrove flats should provide an efficient feeding ground for the Okinawa rail or other birds for a while. On the island of Okinawa, pumice covered a wide area of the coast, which may alter the behavior patterns of various organisms associated with the area. Although the Okinawa rail is not a seabird a recent study reported that migratory birds ingest pumice stones when they were starving^45^. Changes in the behaviors of migratory birds in areas where pumice rafts occupy fishing areas or have been washed ashore may require more attention.

### Impact on local industries and countermeasures of massive pumice stone arrival

The local broadcast stations in Okinawa Prefecture reported that a large amount of pumice aggregated in some fishing harbors in northern Okinawa, advising that pumice stones can damage the propellers of fishing boats and cause engines to overheat. Fishing boats were unable to operate because their drive systems malfunctioned; for example, as the engine coolant system became clogged with pumice, the engine overheated. According to interviews conducted by the Okinawa Prefectural Fisheries Division with local fishing cooperatives, massive pumice stone drifts caused engine trouble in 206 fishing boats (about 7%). In addition, 45 fishing boats were temporarily disabled (about 1.5%) in Okinawa Prefecture in the period up until May 13, 2022^31^. Thus, not only fishing but also the tourism industry is likely to be affected if this situation continues for a very long period. The massive amount of pumice entering enclosed harbours preventing the ready exchange of seawater makes it difficult for the pumice stones to be removed via natural means (Fig. 5, Supplementary Fig. 1b,c), so in some cases removal work was done by heavy machinery. Oil fences were installed to prevent pumice from entering some harbors.

To minimize the impact of pumice rafts on coastal infrastructure such as ports and harbours, it will be necessary to make advances in predicting the movement of pumice rafts as well as to develop countermeasures for future events. Volcanic activity is common in Sakurajima (Kagoshima Prefecture. Because of this situation, the local port has performed a workload analysis of how to remove drifting pumice after the likely event of a major volcanic eruption^63^. Likewise, assessing the effects of pumice rafts will provide valuable information for planning disaster prevention in Okinawa and other areas in the future.

Here, we describe the possibility of pumice stones serving as a nutrient adsorbent material. Nitrate and phosphate from local and industrial wastewater are the main sources of nutrient loading in the marine environment^64,65^, and these compounds induce the reduction of dissolved oxygen in the ocean^66^. Pumice-associated nutrient uptake driven by microbial activity was suggested, after the pumice rafts created on lake Nahuel Huapi and Lake Espejo following the Puyehue-Cordón Caulle (Chile) eruption in June 2011^39^. The possible role of pumice stones in marine biogeochemical cycles is a topic for future research.

## Conclusions

We reported some examples of the early influences of arriving pumice rafts on a broad range of coastal organisms found on coastal beaches, in estuaries, coral reefs, and mangrove forests. In addition to the impact on fishing activities and ship traffic as a navigational hazard, possible future long-term changes in coastal ecosystems may result from the pumice stranding and persistence in coastal environments. Drifting pumice stones are not only useful for dispersing marine organisms but may have other functions, such as a medium for dispersing the microbiome and the possibility of absorbing nutrients from seawater. Pumice has been carried by the Kuroshio Current and has since affected the coastal environments of Kyushu, Shikoku, and Honshu Islands of Japan. Pumice rafts have also recently reached Taiwan, Philippines, and Thailand which are even further away from the source volcano. We hope this report of the situation will be helpful for additional research carried out in Okinawa and other places where pumice stones have been washed ashore, as further investigations are needed to clarify how the large amount of pumice affects both marine and shallow-water environments. Although the peak of pumice drift seems to have passed within the observation period, long-term investigation is necessary.

## Methods

Okinawa Island does not belong to dry climate zone and thus the surround coasts are not classified as arid nor semi-arid coasts^67^ There are no large rivers nearby and the area is not fed by river sediments. The sedimentary coasts bordering Okinawa Island are mainly formed by marine-biogenic material including corals and foraminifera, together with limited contribution of eolian dust^68,69^. Since the pumice rafts were found at Cape Hedo on 16 October 2021, we commenced a survey focusing on the Yambaru region every weekend. Underwater photography at the reef edge was conducted on 14 November 2021. Drone photography (Fig. 5a) was conducted by Okinawa Times Co., Ltd., which is a local media outlet in Okinawa Prefecture. The images in Figs. 5b and c were provided by the Kunigami Fishing Association. A Coolpix w 300 (Nikon Co., Ltd.) was used to take the photographs. The latitude and longitude of the locations where the images were taken are listed in Supplementary Table 1. The shell length measurement of *Lepas* sp. was carried out on pumice that had just washed ashore on the beach. The first samples were taken from seven pumice stones (31 October 2021), and the second collection were from six pumice stones (13 November 2021). Sampling was conducted by walking 200 m of the shoreline boundary looking for wet pumice with *Lepas* attached. Statistical analysis of shell size of them were analyzed with R version 4.0.3 software. Pumice pebbles were collected from a pumice raft at Ibu beach on 15 January 2022. A Leica M165 stereo microscope was used for pumice pebble observation. Pebbles were excited 485/10 nm with ET GFP/CY3 band pass filter. The red autofluorescent signal from algae was detected by a color digital camera (also see Supplemental Fig. 2, 3). The green autofluorescence from pumice, which may have come from the mineral, is minimal or absent under fluorescent microscopy. Adobe premiere pro and image J software were used for video and image editing.

## Supplementary Information

Supplementary Video 1.

Supplementary Video 2.

Supplementary Video 3.

Supplementary Video 4.

Supplementary Video 5.

Supplementary Video 6.

Supplementary Video 7.

Supplementary Video 8.

Supplementary Video 9.

Supplementary Information 1.

## Data Availability

All data generated or analyzed during this study are included in this published article and its supplementary information files.
