# Supplementary material for: Coastal ecological impacts from pumice rafts
Source: Sci Rep. 2022 Jul 19;12:11187. doi: 10.1038/s41598-022-14614-y (PMC9296652; doi:10.1038/s41598-022-14614-y)
Supplement: Supplementary file 10 — Supplementary Information 1. [file 41598_2022_14614_MOESM10_ESM.docx]

Supplementary information

**Coastal ecological impacts from pumice rafts**

Yoshikazu Ohno^1^, Akira Iguchi^2,3^, Mariko Ijima^2^, Ko Yasumoto^1^ ＆Atsushi Suzuki^2,3^

^1^ School of Marine Biosciences, Kitasato University, 1-15-1 Kitasato, Minami, Sagamihara,

Kanagawa 252-0373, Japan

^2^ Geological Survey of Japan, National Institute of Advanced Industrial Science and

Technology (AIST), 1-1-1 Higashi, Tsukuba, Ibaraki 305-8567, Japan

^3^Research Laboratory on Environmentally Conscious Developments and Technologies

[E-code], National Institute of Advanced Industrial Science and Technology (AIST), Tsukuba,

Ibaraki 305-8567, Japan

Correspondence and requests for materials should be addressed to A. I.

Email address: [iguchi.a@aist.go.jp](about:blank)

**Supplementary Figure 1**

**Supplementary Figure 2**

**Supplementary Figure 3**

**Supplementary Table 1**

**Supplementary Video 1**

**Supplementary Video 2**

**Supplementary Video 3**

**Supplementary Video 4**

**Supplementary Video 5**

**Supplementary Video 6**

**Supplementary Video 7**

**Supplementary Video 8**

**Supplementary Video 9**

**Supplementary Figure 1:** View of Oku River, Oku and Ada harbors where the pumice stones washed ashore on northern Okinawa Island
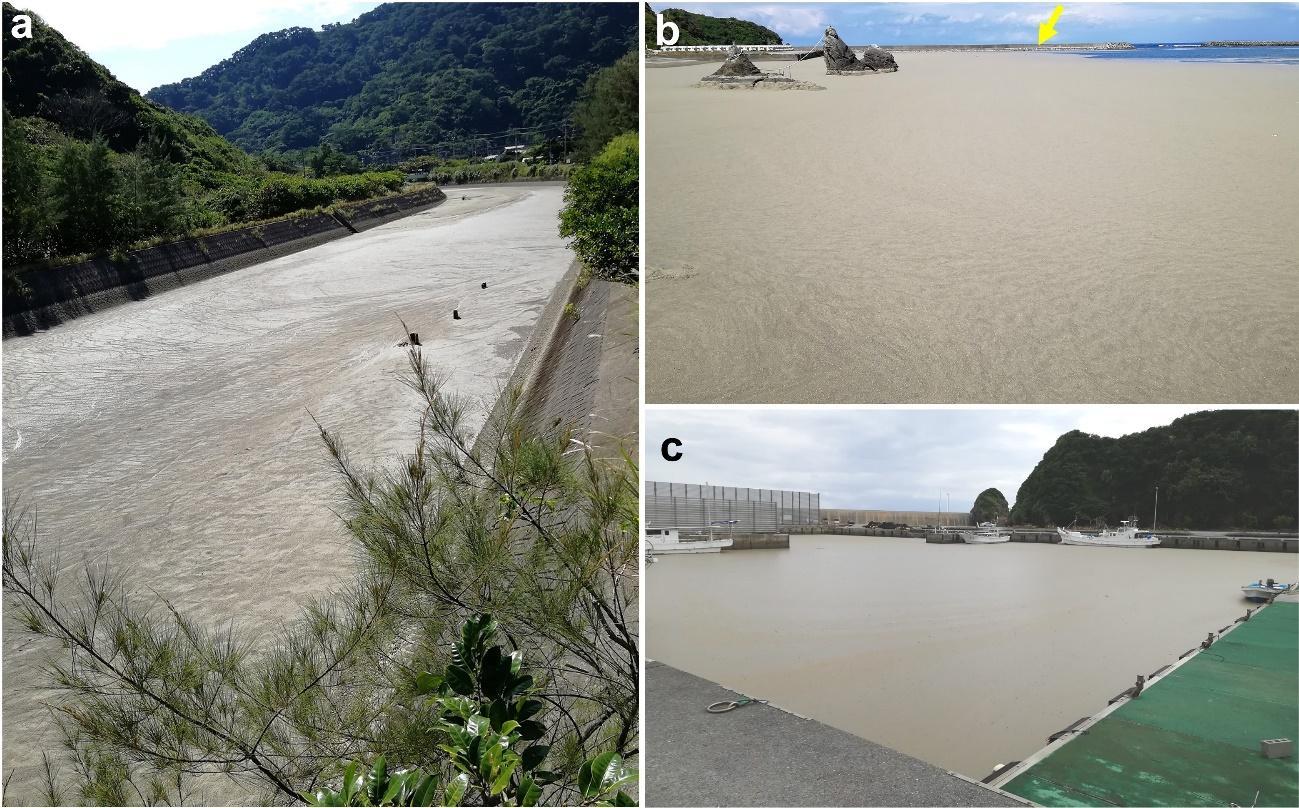


(a) A photo of the Oku River taken looking upstream from a point 200–300 m from the river mouth near the Oku fishing port in (b). The surface of the river between the dikes was almost completely covered with pumice. (b) The pumice-covered Oku fishing harbor is shown, more than 10 km north of (c). The yellow arrow points to the seawall in the bay, approximately 200 m away from the photographer. (c) The fishing boats were surrounded by floating pumice stones and could not be operated on. The thickness of the floating pumice stone layer was approximately 10 cm. The surface was smooth like dried sand, without a slimy feeling when the pumice layer was touched. All photos were taken on 24 October 2021.

**Supplementary Figure 2:** Decrease of fluorescence signal by organic solvent treatment
The upper row (a-c) shows brightfield images corresponding to each, showing fluorescence images (d-e). (a, d) Non-treatment pumice stone sample, but vivid fluorescent signals were detected. (b, e) Pumice stones were treated with 90% of acetone for 10 min at room temperature. (e, f) Treated with 100% of methanol for 10 min at room temperature. All scale bar: 5 mm. The extracts with remaining chlorophyll were washed off and images were taken. A Leica M165 stereomicroscope was used to take these images. Pumice stones were excited 485/10 nm by using ET GFP3/CY3 filter and the signal was detected by a color digital camera. The red fluorescence was clearly reduced, but it was difficult to expunge it from the pumice even after each treatment (e, f), suggesting that the microalgae has invaded the air-trap pores of the pumice pebbles. The green autofluorescence from pumice, which may have come from the mineral, is minimal or absent under fluorescent microscopy.
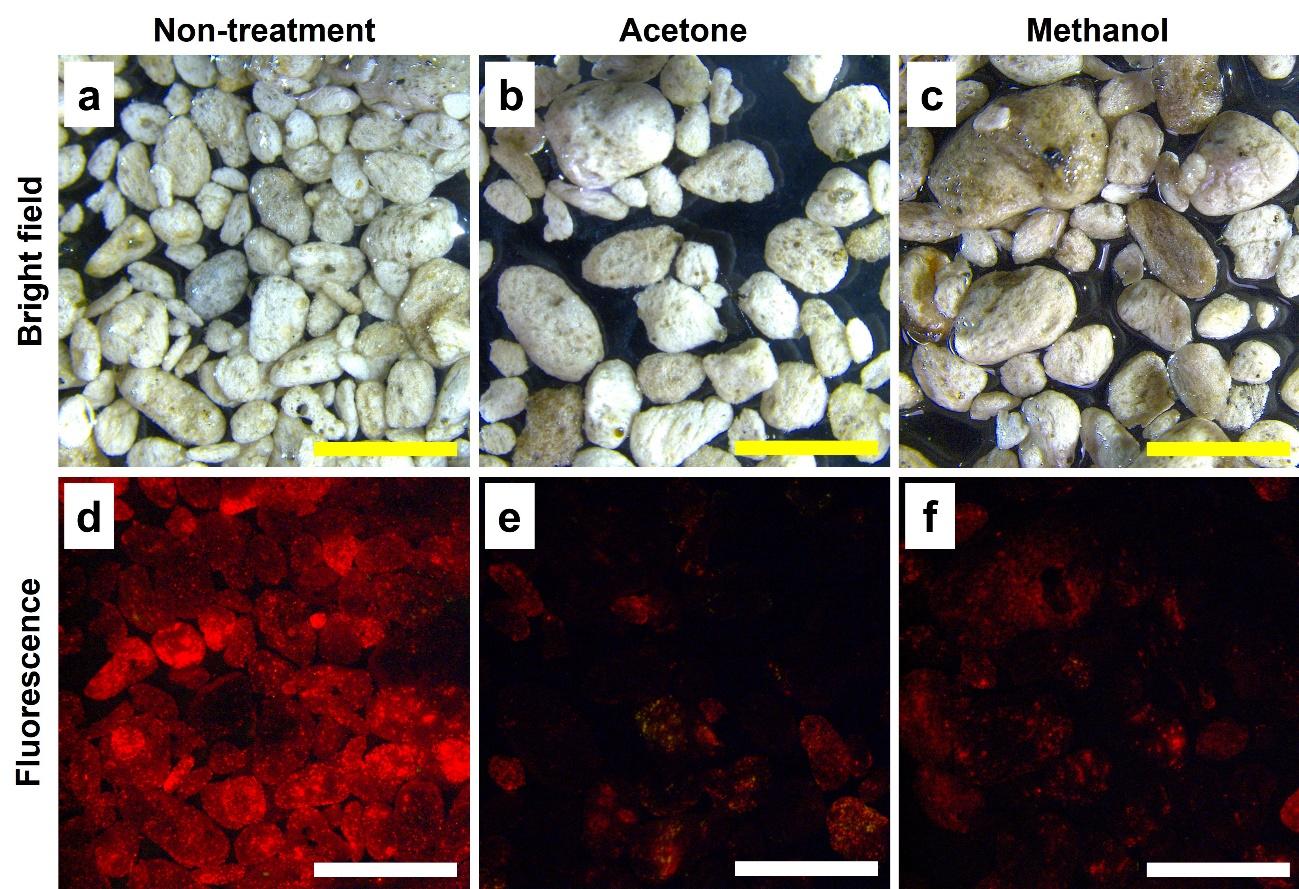


**
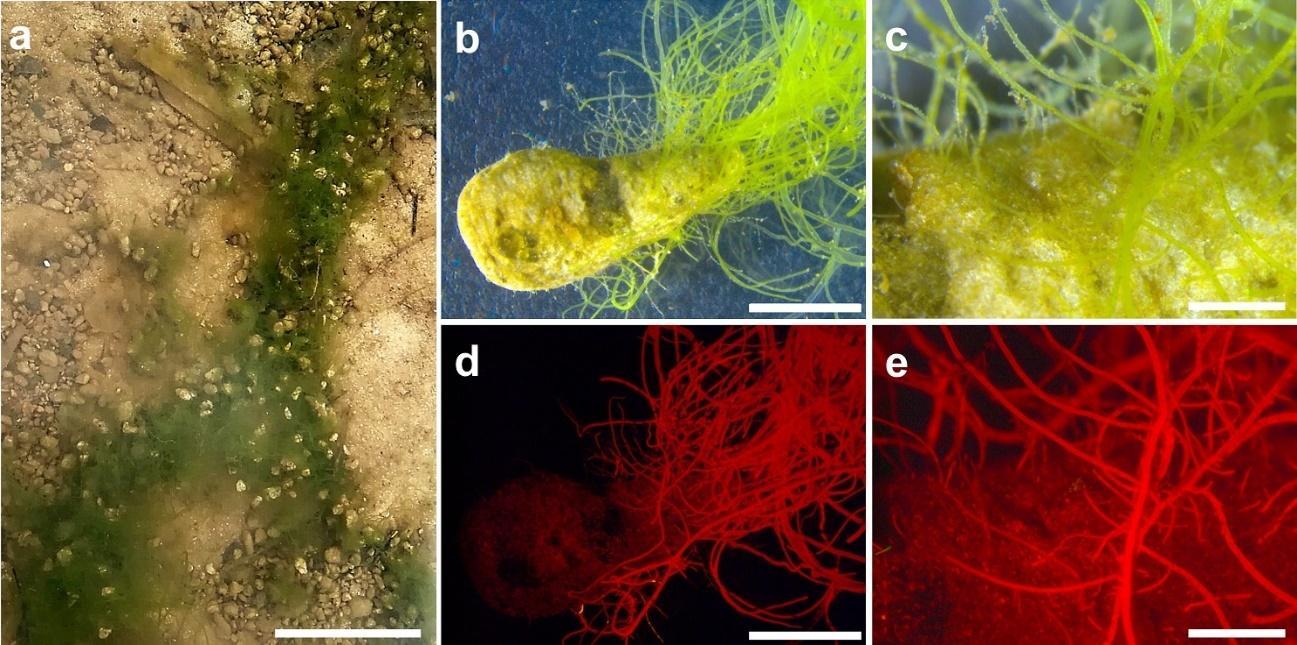
**

**Supplementary Figure 3**: Pumice pebbles covered in benthic filamentous algae
(a) Pumice pebbles on the river bottom are beginning to be covered with filamentous algae. The photo was taken on 15 January 2022 near a brackish water estuary off the Ibu coast (Figure 8). Scale bar 10 cm. (b) Microscopic image of a pumice pebble from (a). Filamentous algae are growing from the pumice surface. Scale bar 5 mm. (c) Enlarged image of the center of the figure of (b) shows not only the filamentous algae but also an area slightly browned in sheet form. Scale bar 1 mm. (d) Chlorophyll fluorescence image corresponds to (b). Scale bar 5 mm. (e) Larger image of (c) matches the shape of algae. Scale bar 1 mm.

**Supplementary Table 1:** Survey areas conducted in Kunigami district

| **Name of place** | **Latitude (N)** | **Longitude (E)** | **Corresponding figure** |
| --- | --- | --- | --- |
| Ibu beach | 26°75′57.9″ | 128°32′23.3" | Fig. 2, Fig. 4  Supplementary Figure 2  Supplementary Video 2 |
| Sate coast | 26°78′84.6″ | 128°22′30.6″ | Fig. 3a and 3b  Supplementary Video 1 |
| Uka coastal area | 26°80′83.3″ | 128°23′38.6″ | Fig. 3c and 3d |
| Hentona fishing harbor | 26°74′83.8″ | 128°17′76.3″ | Fig. 5 |
| Iji coastal area | 26°46'00.2″ | 128°11'35.3" | Fig. 6, Fig. 7  Supplementary Video 3  Supplementary Video 4  Supplementary Video 5 |
| Oku harbor | 26°84′97.4″ | 128°28′99.5″ | Supplementary Figure 1b |
| Ada harbor | 26°74′15.8″ | 128°31′86.2″ | Supplementary Figure 1c |

**Video Legends**

**
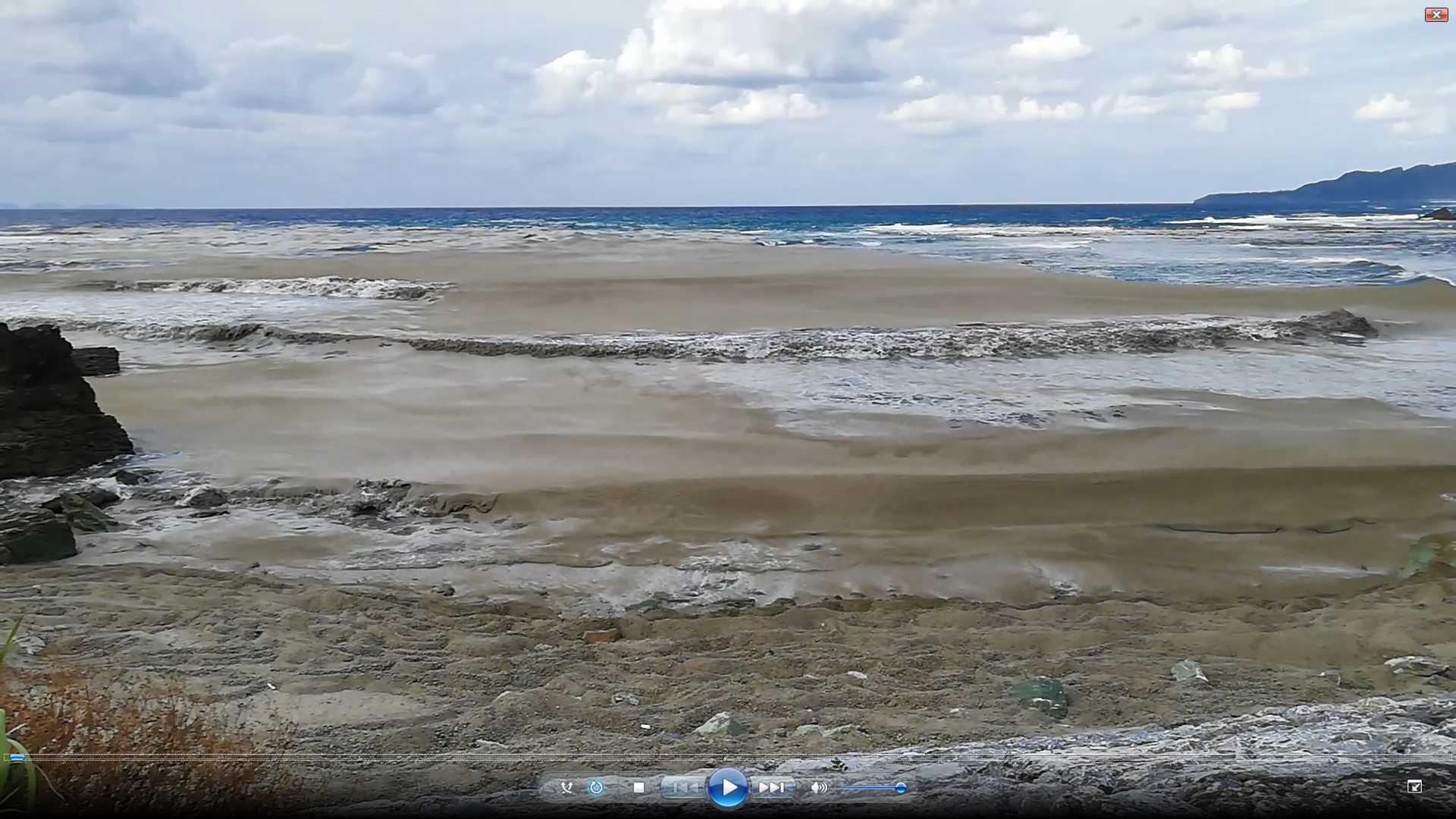
**

**Supplementary Video 1:** Pumice stones being washed ashore by the north wind

This video was taken on 24 October 2021 at the same location as Figure 3a.

**
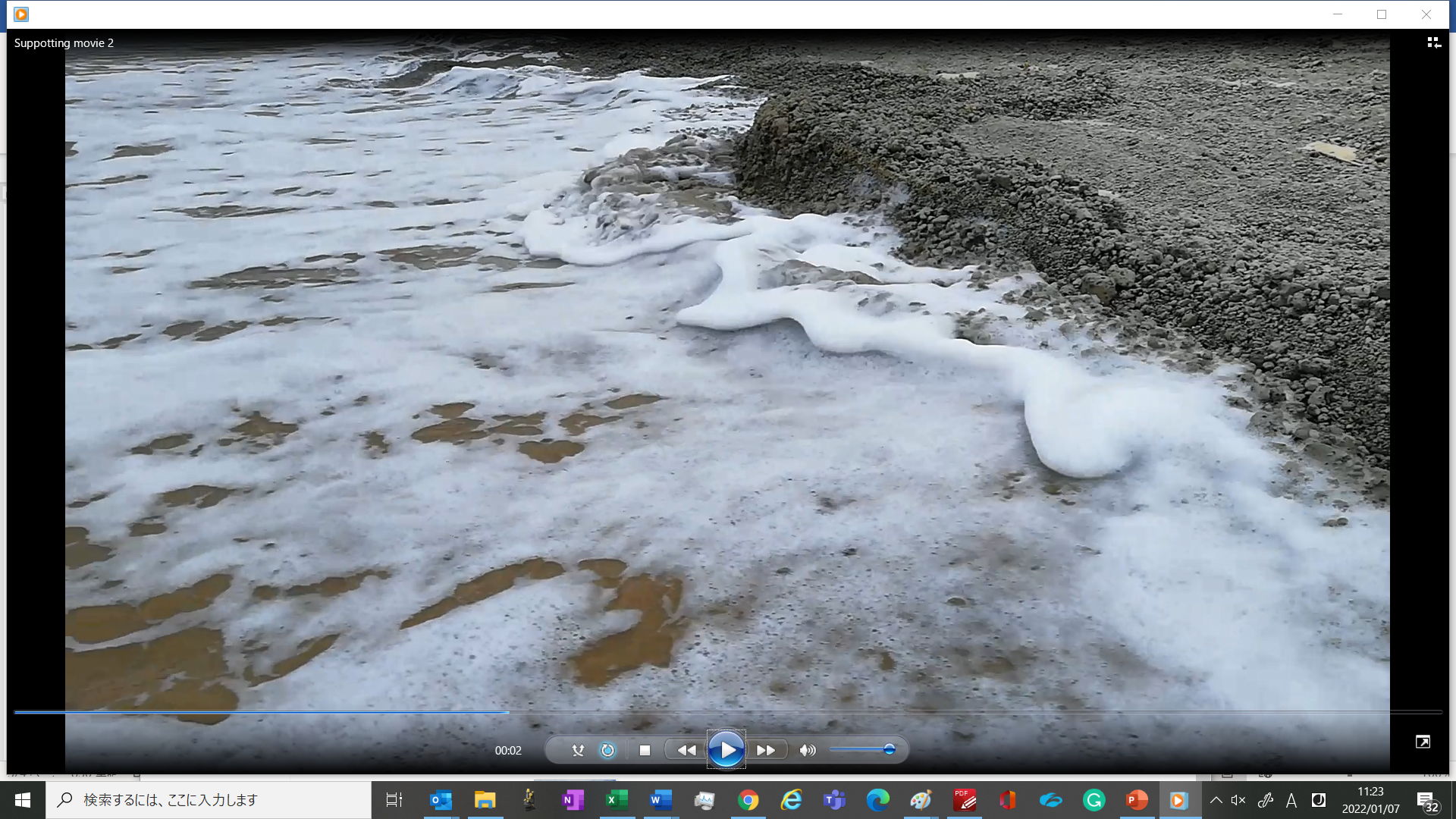
**

**Supplementary Video 2:** A pumice deposit layer collapsing at high tide

A layer of pumice deposited on the shoreline and broken up by the waves. Vigorous wave movement is believed to be necessary to remove pumice from the beach. This video was recorded on Ibu beach on 2 November 2021.

**
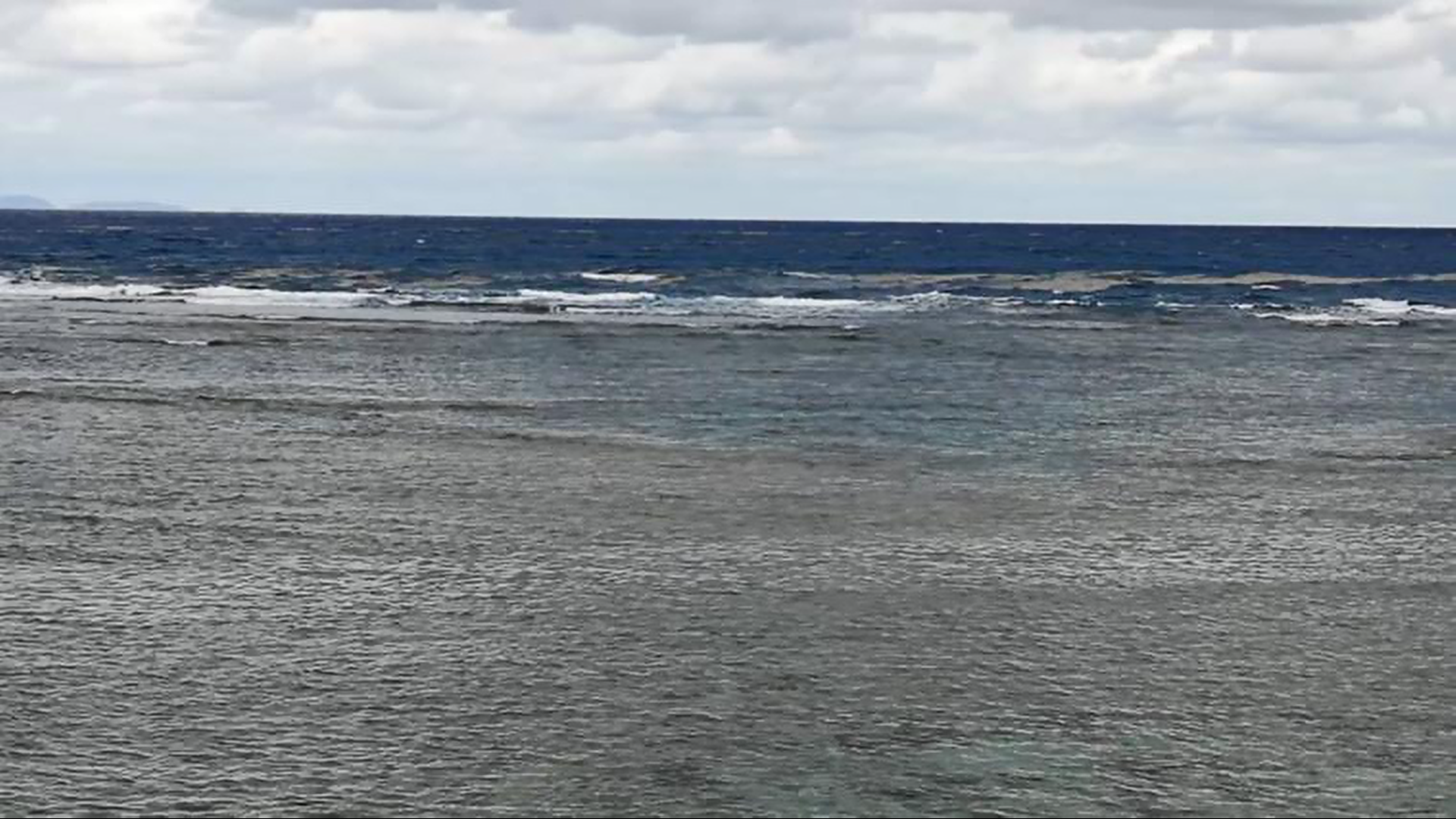
**

**Supplementary Video 3:** Pumice raft washed ashore and smashed by the waves on the reef edge

This video was taken on 14 November 2021. Underwater view is described in Figure 6,


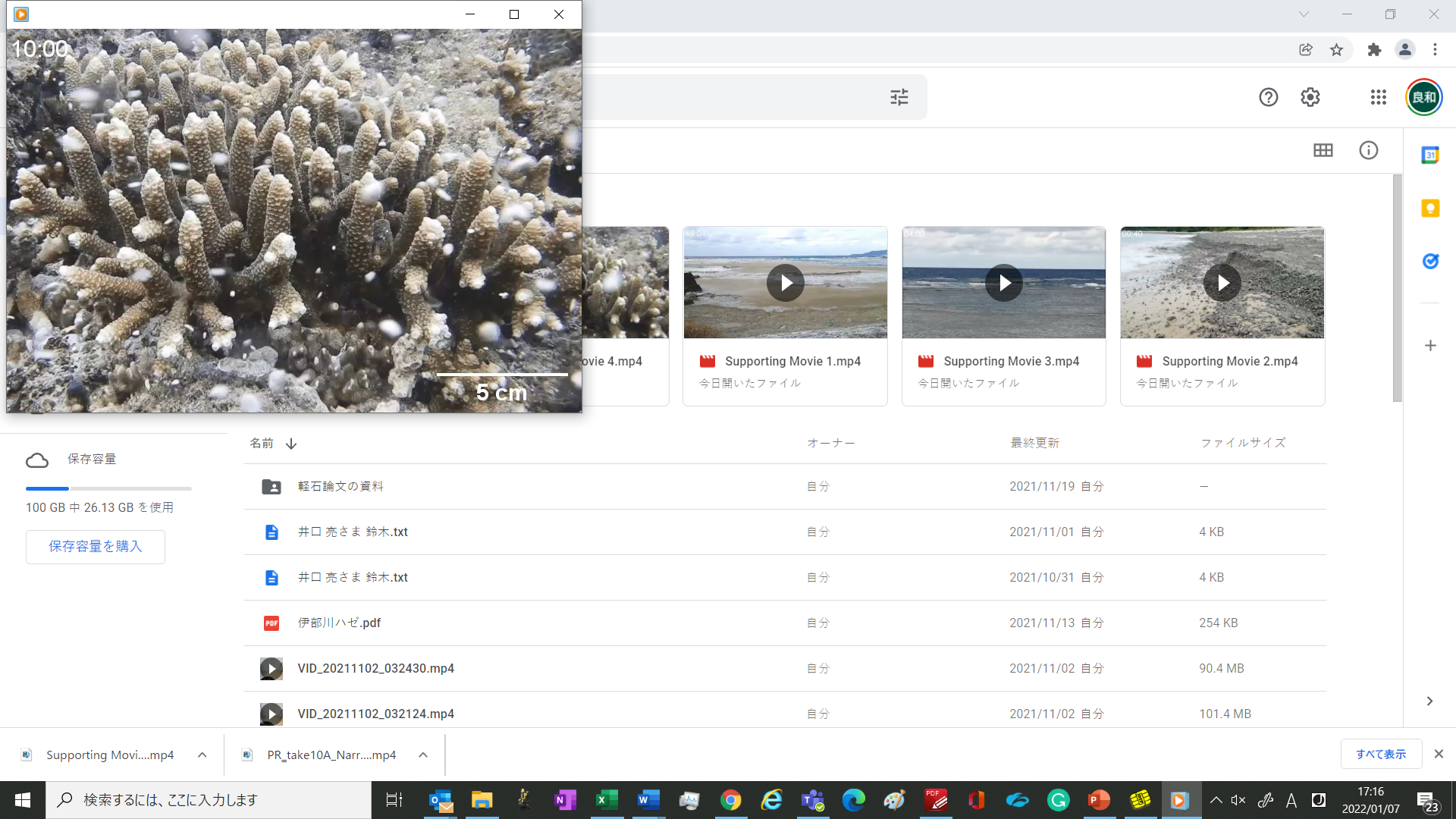


**Supplementary Video 4:** Pumice stones hit corals at the reef edge

Numerous small pumice stones hit *Acropora* coral branches due to wave motion. This image corresponds to Figure 7a.

**Supplementary Video 5:** Pumice stones sunk to the bottom of the river
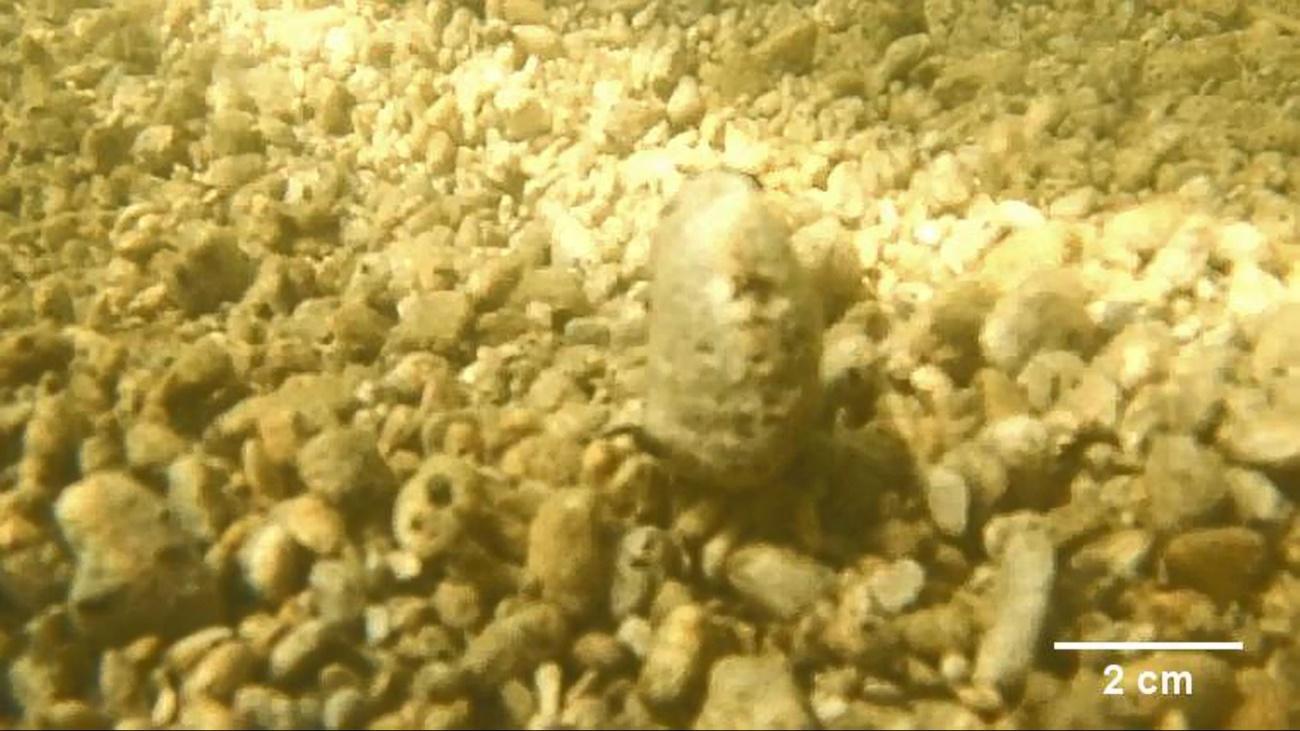


Pumice stones sunk to the bottom of the river and swayed slowly with the current. This video was recorded at the same site as Figure 8b on 2 November 2021. Filamentous algae could not be seen on the pumice surface on that date.


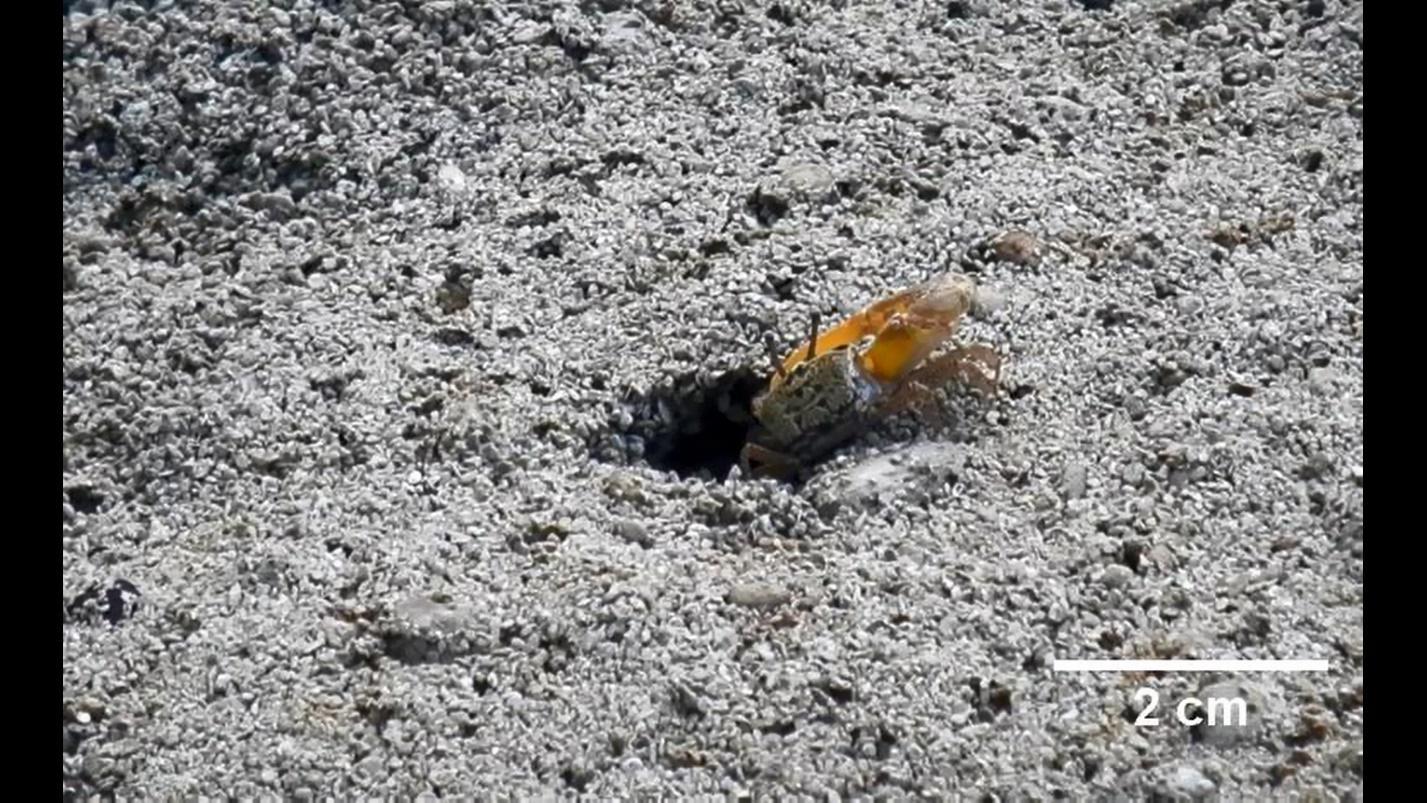


**Supplementary Video 6:** Fiddler crab hesitant to return to the pumice-covered burrow

The layer of small pumice stones is easily broken down, and the opening of the nest is blocked by the pumice stones. One of the male *Uca lactea lacteal* tried twice to enter the burrow by changing the position of its body but the attempt was unsuccessful. This movie was recorded on 21 November 2021.

**Supplementary Video 7:** Intraspecific competition to acquire burrows
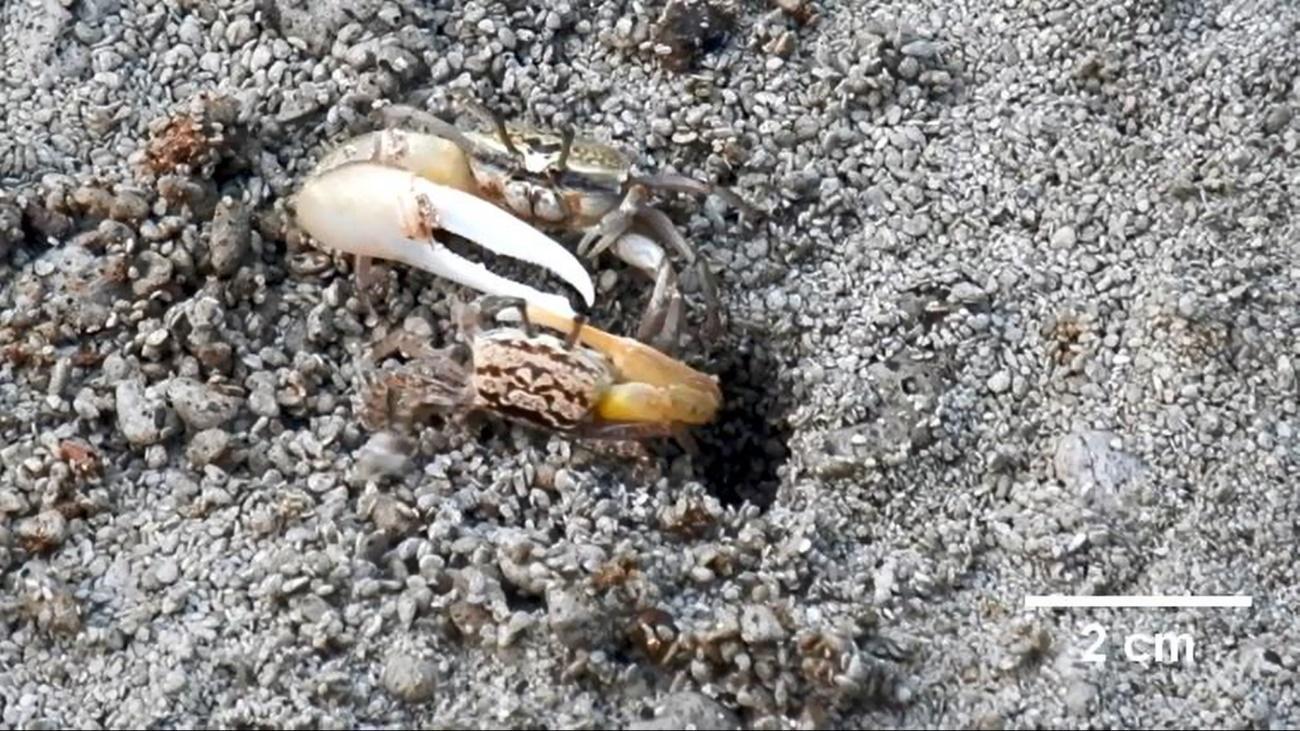


A larger *Uca lactea lacteal* pulls out a smaller crab that was originally in the burrow. The larger crabs began digging their own nests after the smaller crabs were driven out. This video was recorded on 21 November 2021.


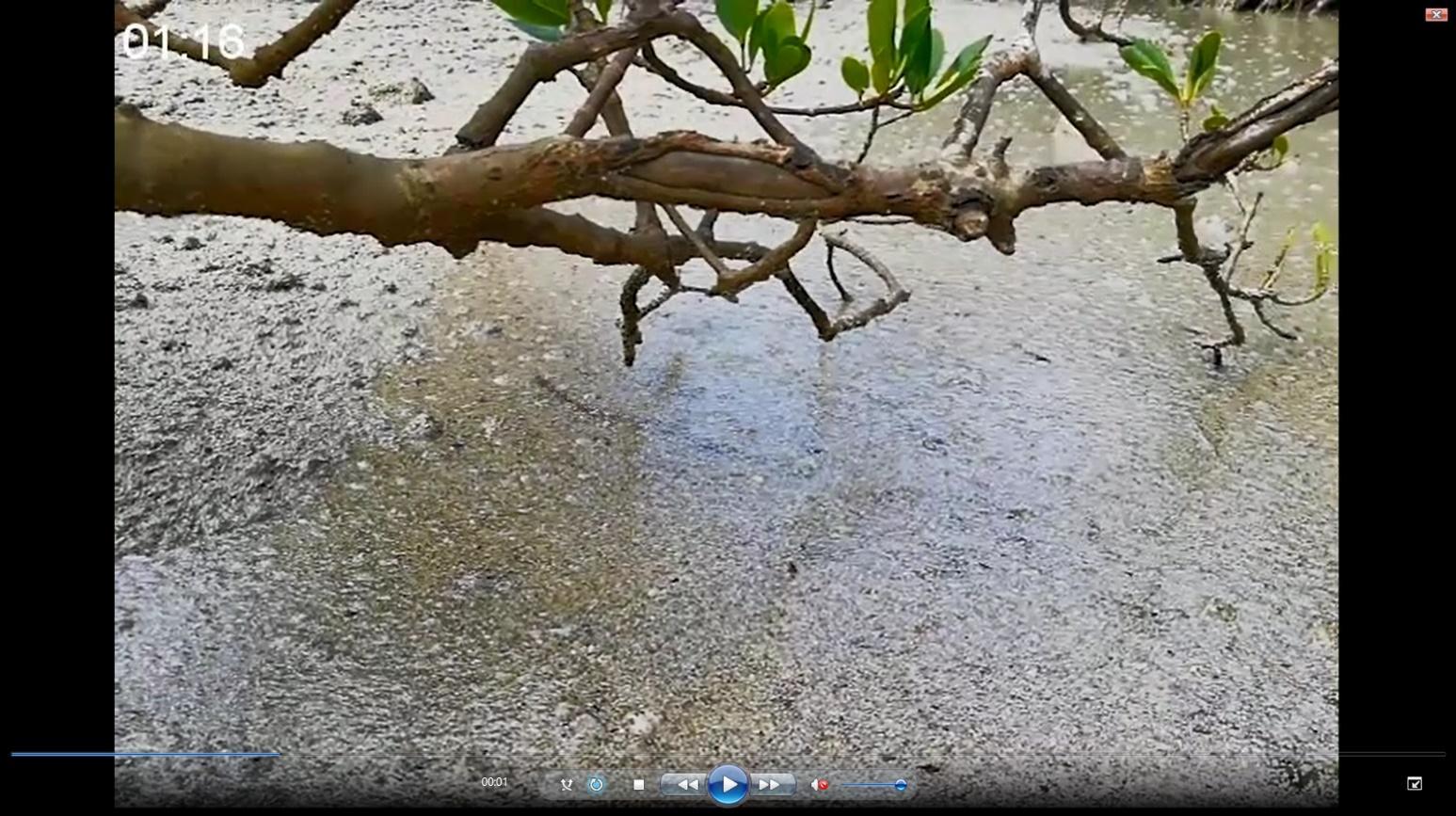


**Supplementary Video 8:** Mudskipper prevented moving properly on pumice-covered water

This video shows that the mudskipper could not bounce and move on the surface of the water properly because the pumice stones covered the water surface. Another goby was trying to poke its head out of the pumice-covered water in the second half of this video. This movie was recorded on 2 November 2021.


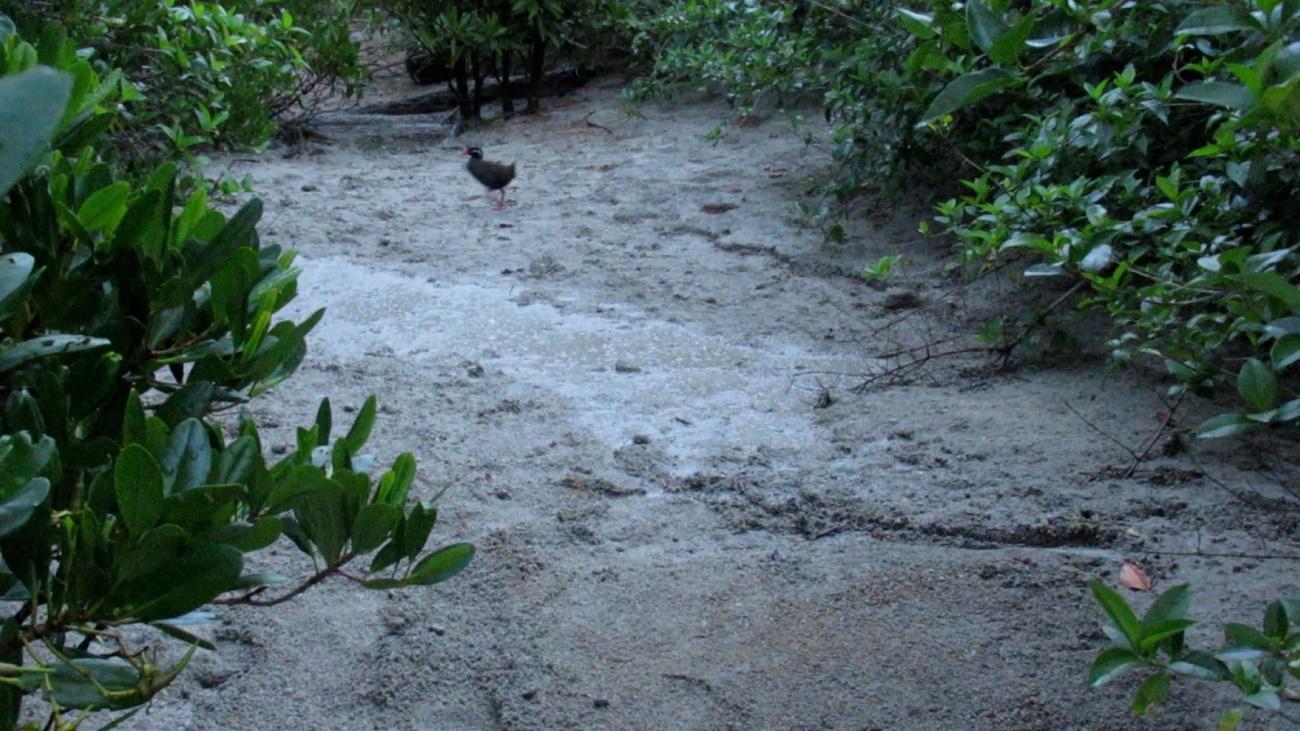
**Supplementary Video 9:** Okinawa rail (*Hypotaenidia okinawae*) crossing a pumice-covered mangrove mudflat

*H. okinawae* cautiously moved over a pumice-covered Ibu mangrove forest early in the morning on 28 November 2021. We could see the bird's characteristic dark red bill and legs. *H. okinawae* is a flightless bird with well-developed legs that sometimes moves very fast, so video playback was carried out at half speed (15 frames per second). *H. okinawae* is categorized as the IUCN Red List of Threatened Species. <https://doi.org/10.2305/IUCN.UK.2016-3.RLTS.T22692412A93352408.en>
